# Differential effects of dopamine signalling on long-term memory formation and consolidation in rodent brain

**DOI:** 10.1186/s12953-015-0069-2

**Published:** 2015-03-18

**Authors:** Nicole Reichenbach, Ulrike Herrmann, Thilo Kähne, Horst Schicknick, Rainer Pielot, Michael Naumann, Daniela C Dieterich, Eckart D Gundelfinger, Karl-Heinz Smalla, Wolfgang Tischmeyer

**Affiliations:** Special Lab Molecular Biological Techniques, Leibniz Institute for Neurobiology, Magdeburg, 39118 Germany; Institute of Experimental Internal Medicine, Medical School, Otto von Guericke University, Magdeburg, 39120 Germany; Department Neurochemistry and Molecular Biology, Leibniz Institute for Neurobiology, Magdeburg, 39118 Germany; Research Group Neuralomics, Leibniz Institute for Neurobiology, Magdeburg, 39118 Germany; Institute for Pharmacology and Toxicology, Medical Faculty, Otto-von-Guericke-University Magdeburg, Magdeburg, 39120 Germany; Center for Behavioral Brain Sciences, Magdeburg, 39106 Germany; Molecular Neurobiology, Medical Faculty, Otto-von-Guericke-University Magdeburg, Magdeburg, 39120 Germany; Present address: Research Group Neurovascular Diseases, German Center for Neurodegenerative Diseases (DZNE), Ludwig-Erhard-Allee 2, Bonn, 53175 Germany; Present address: Division of Cellular Neurobiology, Zoological Institute, TU Braunschweig, Braunschweig, 38106 Germany

**Keywords:** Dopamine agonist, Brain, Cortex, Hippocampus, Striatum, α-synuclein, Auditory discrimination learning, Memory consolidation, Proteome, 2D gel

## Abstract

**Background:**

Using auditory discrimination learning in gerbils, we have previously shown that activation of auditory-cortical D1/D5 dopamine receptors facilitates mTOR-mediated, protein synthesis-dependent mechanisms of memory consolidation and anterograde memory formation. To understand molecular mechanisms of this facilitatory effect, we tested the impact of local pharmacological activation of different D1/D5 dopamine receptor signalling modes in the auditory cortex. To this end, protein patterns in soluble and synaptic protein-enriched fractions from cortical, hippocampal and striatal brain regions of ligand- and vehicle-treated gerbils were analysed by 2D gel electrophoresis and mass spectrometry 24 h after intervention.

**Results:**

After auditory-cortical injection of SKF38393 – a D1/D5 dopamine receptor-selective agonist reported to activate the downstream effectors adenylyl cyclase and phospholipase C – prominent proteomic alterations compared to vehicle-treated controls appeared in the auditory cortex, striatum, and hippocampus, whereas only minor changes were detectable in the frontal cortex. In contrast, auditory-cortical injection of SKF83959 – a D1/D5 agonist reported to preferentially stimulate phospholipase C – induced pronounced changes in the frontal cortex. At the molecular level, we detected altered regulation of cytoskeletal and scaffolding proteins, changes in proteins with functions in energy metabolism, local protein synthesis, and synaptic signalling. Interestingly, abundance and/or subcellular localisation of the predominantly presynaptic protein α-synuclein displayed dopaminergic regulation. To assess the role of α-synuclein for dopaminergic mechanisms of memory modulation, we tested the impact of post-conditioning systemic pharmacological activation of different D1/D5 dopamine receptor signalling modes on auditory discrimination learning in α-synuclein-mutant mice. In C57BL/6JOlaHsd mice, bearing a spontaneous deletion of the α-synuclein-encoding gene, but not in the related substrains C57BL/6JCrl and C57BL/6JRccHsd, adenylyl cyclase-mediated signalling affected acquisition rates over future learning episodes, whereas phospholipase C-mediated signalling affected final memory performance.

**Conclusions:**

Dopamine signalling modes via D1/D5 receptors in the auditory cortex differentially impact protein profiles related to rearrangement of cytomatrices, energy metabolism, and synaptic neurotransmission in cortical, hippocampal, and basal brain structures. Altered dopamine neurotransmission in α-synuclein-deficient mice revealed that distinct D1/D5 receptor signalling modes may control different aspects of memory consolidation.

**Electronic supplementary material:**

The online version of this article (doi:10.1186/s12953-015-0069-2) contains supplementary material, which is available to authorized users.

## Background

New memories consolidate over time from an initially labile state to a more permanent state (for review, see, *e.g.* [1-3]). Long-term memory formation is thought to depend on long-lasting alterations in cerebral neurons and, in particular, in the efficacy of their synaptic connections, involving structural rearrangements of synapses. At the systems level, concepts of memory consolidation assume an active redistribution of memory representations from temporary into long-term stores [4], involving interactions of networks in cortical and more basal brain regions over days or weeks.

Current views of the role of synaptic plasticity in memory formation involve, in addition to memory-stabilising mechanisms, processes that improve the ability for long-lasting plastic reassembly of neurons and synapses [5-7]. Both permissive and stabilising processes are likely to require *de novo* protein synthesis and alterations at the posttranslational level, including the modification, localisation, and degradation of proteins [8-10]. Signalling pathways that control cerebral protein metabolism are, therefore, likely to be involved in the regulation of synaptic plasticity underlying long-term memory formation. Neuromodulators, such as dopamine, have been implicated in the regulation of synaptic plasticity and translation and in the consolidation of memory traces [11,12].

The auditory cortex (AC) is critical for learning the discrimination of the directions of modulation (rising *vs.* falling) of linearly frequency-modulated tones (FMs) [13-15]. As shown for Mongolian gerbils, long-term memory formation in this paradigm requires post-acquisition protein synthesis in the AC. Moreover, inhibitors of protein synthesis and of mammalian target of rapamycin (mTOR), a protein kinase implicated in the control of synaptic plasticity and translation [16], interfere with long-term memory formation (but not with acquisition or short-term memory) for a number of training days when applied to the AC shortly after the initial conditioning to FMs [17,18]. This implies that auditory discrimination learning induces a protein synthesis-dependent signal in the AC that prepares local circuits and/or distributed networks for memory formation in future learning episodes. Accordingly, after FM discrimination learning in mice, adaptive synaptic proteome changes supposed to facilitate long-lasting plastic rearrangements were monitored in the AC as well as in frontal cortical, hippocampal, and striatal regions [19] known to maintain direct or indirect connections with the AC [20].

The gerbil AC receives projections from the dopaminergic midbrain [20] and displays D1 dopamine receptor immunoreactivity [21]. Increased cortical dopamine release during and shortly after conditioning of gerbils to FMs is critical for the establishment of this complex behaviour [22-24]. Thus, dopamine is likely to participate in the regulation of mechanisms that control long-term memory formation in this learning paradigm. Accordingly, SKF38393, an agonist of the class of D1-like dopamine receptors (*i.e*., D1 and D5 receptors), substantially improved memory consolidation when administered locally into the AC shortly after differential conditioning of gerbils to FMs. Moreover, the dopamine agonist efficiently enhanced memory consolidation even when applied one day in advance of the initial learning event; inhibitors of protein synthesis and mTOR prevented this effect [21]. Together, these findings suggest that D1/D5 dopamine receptor activation in the AC may induce mTOR-mediated, protein synthesis-dependent changes in the gerbil brain that persist for at least 24 h and facilitate the consolidation of FM discrimination memory.

In search of molecular components involved in this memory-enhancing effect observed in gerbils, the aim of the present study was to identify proteins differentially regulated in selected brain regions of naïve gerbils after artificial pharmacological activation of auditory-cortical D1/D5 dopamine receptors. To this end, protein profiles of two fractions, *i.e.*, a triton-soluble protein (TP) fraction and a synaptic junctional protein-enriched (SP) fraction, obtained from the AC, frontal cortex (FC), hippocampus (HC), and striatum (ST) of gerbils 24 h after intra-AC injection of D1/D5 dopamine receptor selective agonists were compared with the corresponding protein profiles of vehicle-treated control gerbils using 2D gel electrophoretic separation of proteins in conjunction with mass spectrometry to identify tryptic peptides of differentially regulated protein spots. Two quantitative proteomic screens were performed in an analogous manner, utilising benzazepine D1/D5 dopamine receptor agonists of different effector selectivity. In the first screen, SKF38393 was used, which was reported to induce the activation of both known downstream effectors of D1/D5 dopamine receptors, *i.e*., adenylyl cyclase (ADCY) and phospholipase C (PLC) [25]. To gain insight into initial signalling modes by which auditory-cortical D1/D5 receptors might control memory-relevant proteome changes, a second screen was performed utilising SKF83959. This agonist was reported to antagonise dopamine-mediated stimulation of ADCY and to preferentially activate PLC [26,27] (but see [28]). Differential regulation of selected proteins was validated by immunoblot analysis and/or immunocytochemical studies on primary cultured neurons. Finally, behavioural experiments were performed to study the role of one of the differentially regulated proteins, the predominantly presynaptic protein α-synuclein, for FM discrimination learning and its dopaminergic modulation. These studies were performed on a genetic mouse model as α-synuclein deficient gerbils are not available.

## Results

### Proteome analysis

To detect proteome changes in the gerbil brain associated with pharmacological activation of auditory-cortical D1/D5 dopamine receptors, 2D gel electrophoresis was employed to compare protein abundances in Triton-soluble (TP) and Triton-insoluble (enriched for synaptic junctional proteins; SP) protein fractions from the AC, FC, HC, and ST 24 h after intra-AC infusion of SKF38393 or SKF83959 with those of vehicle-infused control counterparts. Silver-stained 2D gels were scanned with a calibrated imaging densitometer. Quantification and differentially regulated spot selection were performed using PDQuest software. Proteins in significantly regulated spots were identified by nanoLC-ESI-tandem mass spectrometry.

In the analysed brain regions of SKF38393-treated gerbils, a total of 167 protein spots, representing 404 identified proteins (*i.e*., 240 in up- and 164 in down-regulated spots), differed significantly from the corresponding spots of vehicle-treated controls. After SKF83959 treatment, 119 differentially regulated spots were recognised, representing 347 identified proteins (*i.e*., 161 in up- and 186 in down-regulated spots). Note that multiple proteins present in a single spot are not necessarily regulated in the same direction.

**Figure 1:**
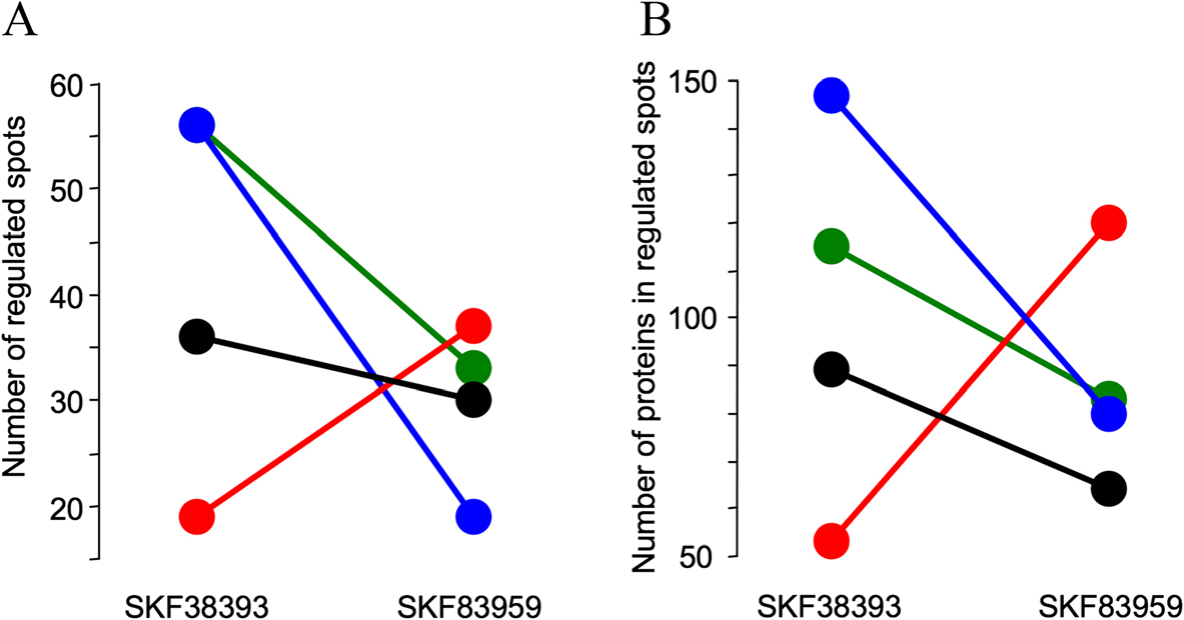
**Regional differences in dopamine agonist-induced proteome changes.** Gerbils (*n* = 6 per group) received bilateral local injections of SKF38393, SKF83959, or vehicle into the auditory cortex and were decapitated 24 h later. Soluble and synaptic junctional protein-enriched fractions prepared from the auditory cortex (blue), frontal cortex (red), hippocampus (black), and striatum (green) were analysed using 2D gel electrophoresis in conjunction with mass spectrometry. Selection of differentially regulated spots in comparison with corresponding vehicle-treated controls (*P* < 0.05, Mann-Whitney’s *U*-test) was performed using image analysis PDQuest software. **A**. Total numbers of all the spots that were regulated per brain region. **B**. Total numbers of proteins identified in all the spots that were regulated per brain region.

Differences in the proteomes in comparison to vehicle-treated controls were detectable in all brain regions analysed after intra-AC agonist treatments. Interestingly, the locally applied agonists – reported to differ in their D1/D5 dopamine receptor-mediated stimulation of downstream effectors – induced differential changes in distinct brain regions (Figure 1). After infusion of SKF38393, most prominent changes were monitored in the target region of injection itself (the AC), and in the ST, whereas the FC exhibited less changes. In contrast, 24 h after intra-AC SKF83959 infusion, the most prominent changes were detectable in the FC.

**Figure 2:**
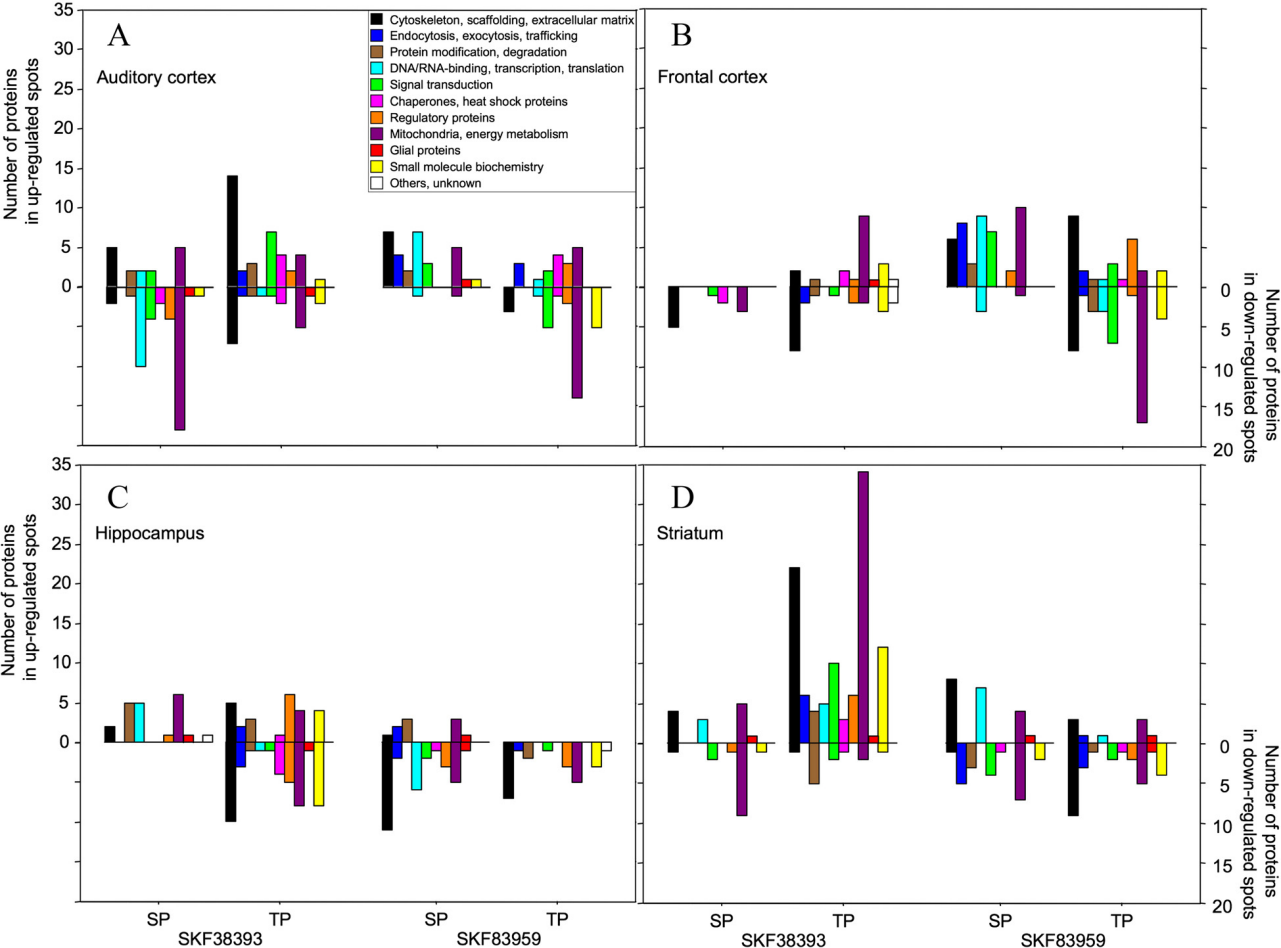
**Regional and functional differences in dopamine agonist-induced proteome changes.** Proteins identified in all differentially regulated 2D gel spots that were obtained from the auditory cortex **(A)**, frontal cortex **(B)**, hippocampus **(C)**, and striatum **(D)** of gerbils 24 h after D1/D5 dopamine receptor agonist injections into the auditory cortex (for details, see legend of Figure 1) were assigned to functional categories on the basis of SynProt (http://www.synprot.de; [29]) as indicated in the inset in panel A. Shown are the numbers of proteins identified in all the spots that were regulated, specified according to agonist, protein fraction, functional category, and direction (up *vs.* down) of spot regulation.

Assigning the proteins identified in differentially regulated spots to functional categories on the basis of the database SynProt (http://www.synprot.de; [29]) revealed that members of the categories “mitochondria, energy metabolism” and “cytoskeleton, scaffolding, extracellular matrix” were most frequently detected after treatment with SKF38393 or SKF83959. The numbers of proteins identified in up- *vs.* down-regulated spots are documented in Figure 2 according to brain region, agonist, protein fraction, and functional category. (Additional file 1: Table S1) gives an overview of the proteins identified in differentially regulated spots, itemised by brain region, agonist, protein fraction, and functional category. Note that in Additional file 1: Table S1 data are partially simplified for reasons of clarity. More detailed information on individual proteins identified in differentially regulated spots are provided in (Additional file 2: Table S2).

Differentially regulated protein spots of the SP fraction may include components of plastic rearrangements of synapses. After SKF38393 treatment, most proteins in such spots obtained from the AC, in particular proteins of the categories “mitochondria, energy metabolism” and “DNA/RNA-binding, transcription, translation”, were detected in down-regulated spots (Figure 2A). In contrast, in the SP fraction from the HC, proteins of these categories were detected exclusively in up-regulated spots (Figure 2C). Indeed, several DNA/RNA-binding proteins in the SP fraction, such as the heterogeneous nuclear ribonucleoproteins (hnRNPs) K and L, were detectable in down-regulated spots from the AC but in up-regulated spots from the HC, whereas no member of this category was regulated in the SP fraction from the FC (*cf.* Additional file 1: Table S1). After SKF83959 treatment, the number of proteins identified in regulated spots of the SP fraction from the AC was smaller than after SKF38393 treatment, but the majority of them was detected in up-regulated spots (Figure 2A). This applied in particular to proteins of the category “DNA/RNA-binding, transcription, translation”. Interestingly, this category includes proteins that were enriched in the SP fraction from the HC after SKF38393 treatment but in the SP fraction from the FC after SKF83959 treatment (*cf.* Figure 2B, C and Additional file 1: Table S1).

In the TP fraction, most prominent changes were monitored in the ST after SKF38393 treatment (Figure 2D). Here, most proteins – in particular those of the categories “mitochondria, energy metabolism” and “cytoskeleton, scaffolding, extracellular matrix” – were identified in up-regulated spots.

To summarise, intra-AC injection of SKF38393 and SKF83959 induced differential changes in protein profiles of the AC itself and of distant frontal-cortical, hippocampal, and striatal regions of the gerbil brain when compared to vehicle-treated controls. Extent and direction of alterations induced by these D1/D5 agonists of distinct effector selectivity differed, in part, among brain regions. Functional categorisation of proteins identified in differentially regulated spots implied that the changes concerned primarily proteins of mitochondria and energy metabolism, and cytoskeleton and scaffolding components. Components of other functional categories, including those with putative functions in inter- and intracellular communication, such as “endocytosis, exocytosis, trafficking”, “DNA/RNA-binding, transcription, translation”, and “signal transduction”, were recognised as well, but to a lesser extent.

### Immunoblot analysis

**Figure 3:**
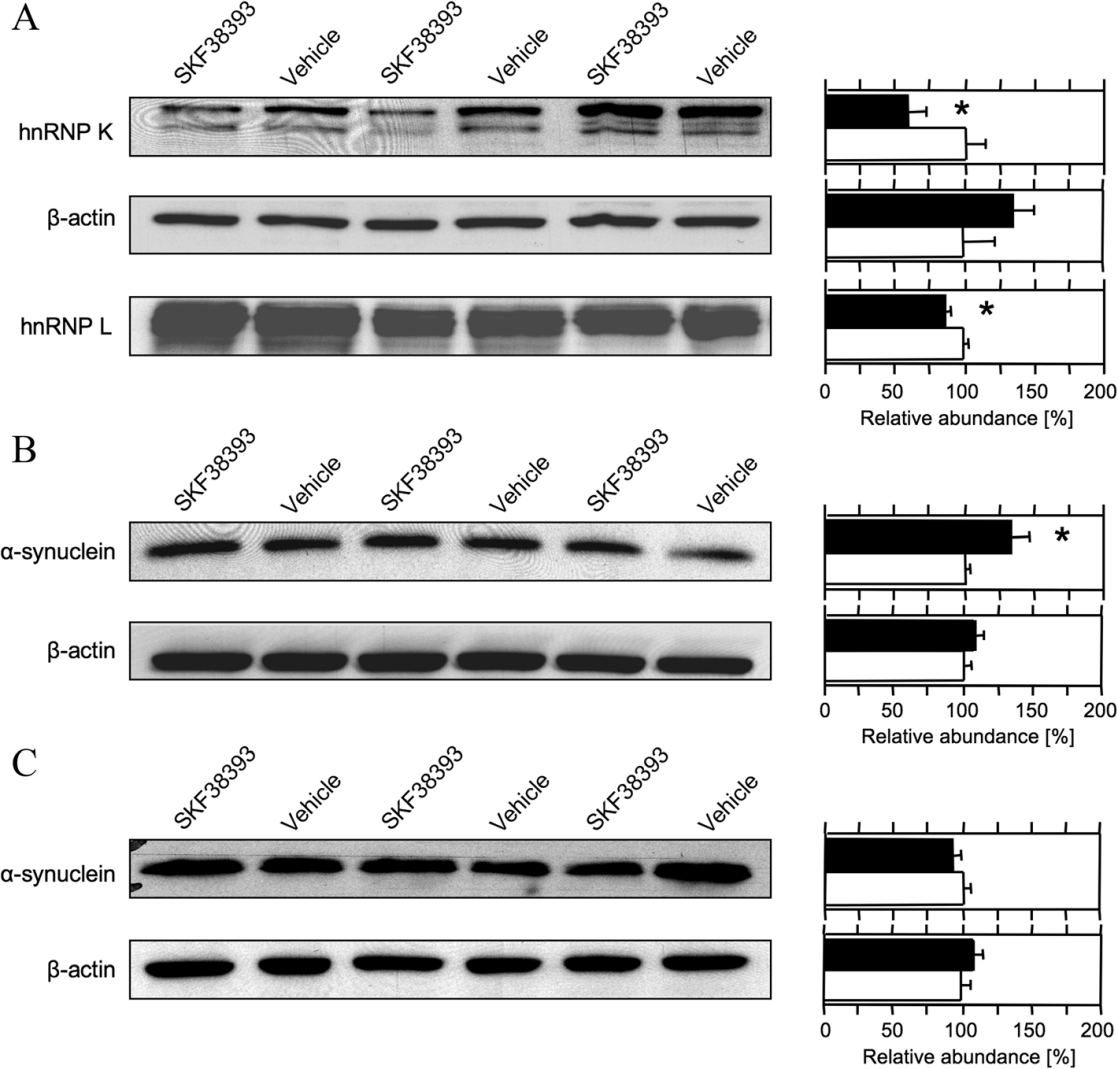
**Quantitative immunoblot analysis of selected proteins in SKF38393- and vehicle-treated gerbils.** Gerbils received bilateral local injections of SKF38393 or vehicle into the auditory cortex and were decapitated 24 h later. **A**. Western blots of the synaptic junctional protein-enriched fraction from auditory cortex probed with antibodies against hnRNP K, β-actin, and hnRNP L. **B**. Western blot of the triton-soluble protein fraction from auditory cortex probed with antibodies against α-synuclein and β-actin. **C**. Western blot of the triton-soluble protein fraction from hippocampus probed with antibodies against α-synuclein and β-actin. Left: immunoblots with ECL signals. Right: optical densities of ECL signals from SKF38393-treated gerbils (filled bars) relative to those from vehicle-treated controls (empty bars). Means + S.E.M.; *n* = 3–6 per group; *P < 0.05 significantly different from controls (Mann–Whitney *U* test).

Proteomic results were validated exemplarily by immunoblot analysis of protein preparations from separate sets of gerbils 24 h after intra-AC injection of SKF38393 or vehicle. Specific antibodies against three proteins reported to be involved in the modulation of synaptic transmission and plasticity were selected, namely the RNA-binding proteins hnRNP K and hnRNP L and the predominantly presynaptic protein α-synuclein. In accordance with the proteomic analysis (*cf*. Additional file 1: Table S1), significant down-regulation of hnRNP K and hnRNP L was monitored in immunoblots of the SP fraction from the AC (Figure 3A). Similarly, the SKF38393-induced up-regulation of α-synuclein that was detected in the TP fraction from the AC by proteomic analysis (*cf*. Additional file 1: Table S1) was confirmed by immunoblot analysis as well (Figure 3B). Notably, no changes in α-synuclein were detected in brain regions other than the AC (*cf*. Additional file 1: Table S1, Figure 3C), that is, the target region of SKF38393 injection. This implies that dopaminergic regulation of this protein in cerebral neurons is mediated locally by agonist-receptor interaction. Therefore, immunocytochemical studies on primary cultured cerebral neurons were performed to further examine the regulation of α-synuclein, in particular, of its subcellular localisation, by SKF38393 (see below).

### Immunocytochemical analysis

The nerve terminal-enriched protein α-synuclein has been implicated, among others, in the modulation of neurotransmitter release [30,31]. Despite its association with synaptic vesicles, α-synuclein is highly mobile and may disperse from the nerve terminal in response to neural activity [32,33]. Therefore, we examined effects of SKF38393 on the localisation of α-synuclein relative to bassoon, a scaffolding protein of the cytomatrix at the active zone of neurotransmitter release [34]. Cultures of 21 days *in vitro* (DIV21) rat hippocampal neurons were incubated with or without SKF38393, fixed, stained with bassoon and α-synuclein antibodies, and visualised using confocal laser scanning microscopy.

**Figure 4:**
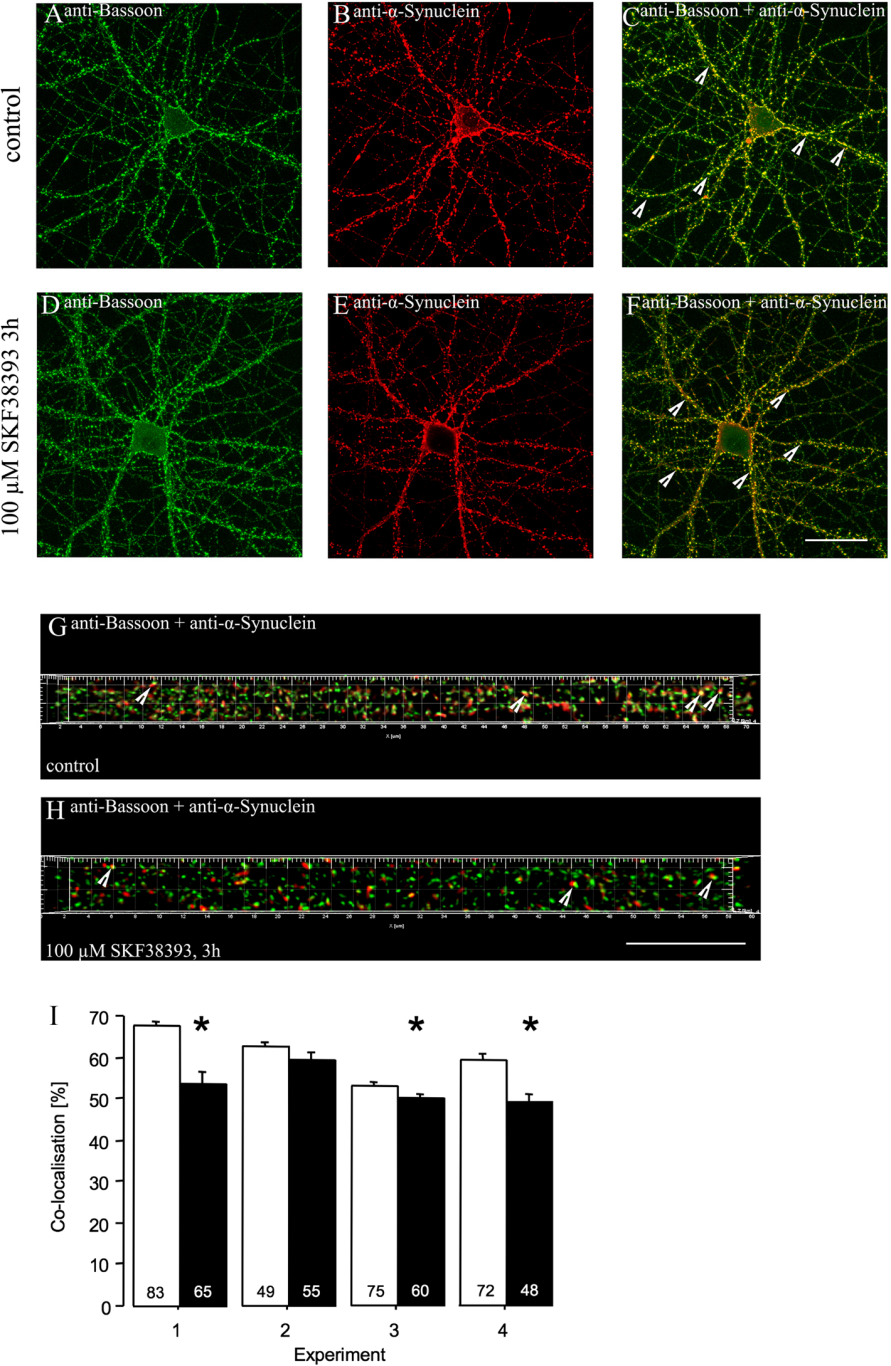
**Immunocytochemical analysis of bassoon and α-synuclein co-localisation in control- and SKF38393-treated primary hippocampal neurons in culture.** In four independent experiments, cultures of DIV21 rat hippocampal neurons were incubated for 3 h in replaced medium containing either no SKF38393 (A-C, control-treatment) or 100 μM SKF38393 (D-F), fixed, stained with bassoon and α-synuclein antibodies, and visualised using confocal laser scanning microscopy. **A-F**. Representative photomicrographs of neurons stained with bassoon (A, D) and α-synuclein (B, E) immunofluorescence and the merged pictures (C, F) (scale bar: 20 μm). **G-H**. Merged pictures of bassoon (green) and α-synuclein (red) immunofluorescence signals obtained from dendrites of control- (G) and SKF38393-treated neurons (H) after straightening and deconvolution for co-localisation analysis (scale bar: 10 μm). Arrows in C, F-H paradigmatically indicate co-localisation of bassoon and α-synuclein. **I**. Co-localisation of bassoon and α-synuclein immunofluorescence signals was quantified in dendrites of ten neurons for each treatment condition from four independent experiments (empty bars: control-treatment; filled bars: SKF38393-treatment). The percentage of bassoon signal co-localised with α-synuclein signal is shown as means + S.E.M. Numbers inside bars are numbers of neuronal dendrites examined. Statistical evaluation was performed using a 2 x 4 (pharmacological treatment x experiment) analysis of variance (ANOVA) and Student’s two-sided *t*-test for unpaired comparisons. ANOVA values are described in the text. *P < 0.05, significantly different from controls (unpaired *t*-test).

Representative confocal images of control- and SKF38393-treated neurons stained for bassoon and α-synuclein are shown in Figures 4A-F. Arrows in the merged pictures exemplarily indicate co-localisation of the two proteins. In Figures 4G-H, merged pictures of bassoon and α-synuclein immunofluorescence signals obtained from synapses on dendrites of control- and SKF38393-treated neurons are shown after straightening and deconvolution for localisation analysis.

Four independent experiments were performed analogously for localisation analysis. Co-localisation of α-synuclein with bassoon immunofluorescence signals was examined in dendrites of 10 neurons per experiment and pharmacological treatment condition. As shown in Figure 4I, the degree of co-localisation of bassoon and α-synuclein signals is on average lower in SKF38393-treated dendrites than in control-treated dendrites in each of the 4 experiments. Evaluation of these measures with a 2 x 4 (pharmacological treatment x experiment) analysis of variance (ANOVA) revealed significant main effects of the factors treatment (*F*1,499 = 49.39; *P* < 0.0001) and experiment (*F*3,499 = 19.14; *P* < 0.0001) and a significant treatment x experiment interaction (*F*3,499 = 6.41; *P* < 0.0005). The main effect of treatment indicates a significant difference in the co-localisation of α-synuclein with bassoon signals between control- and SKF38393-treated neurons. The effect of experiment may be due to variations in experimental parameters, such as neuronal cultures, media or drugs, among the four independent, sequentially performed experiments. The finding that the interaction between the factors treatment and experiment reached statistical significance might point to variations in the expression of the agonist effect on protein localisation in the four experiments. Indeed, comparing the co-localisation data between treatment groups within each of the individual experiments revealed significant group differences in experiments 1, 3, and 4, whereas in experiment 2 this difference did not reach statistical significance (*P* = 0.08).

Co-immunostaining for PSD95 was used to distinguish the postsynaptic compartment from the presynaptic terminal of the synapse, thus serving as a negative control for co-localisation analysis. Notably, the degree of co-localisation of bassoon–α-synuclein positive puncta with PSD95 signal is lower than that of the co-localised signals of bassoon and α-synuclein (Additional file 3: Figure S1); same holds true for the Pearson correlation coefficients (bassoon/α-synuclein: 0.189 ± 0.005; bassoon–α-synuclein/PSD95: 0.107 ± 0.004).

Together, these findings in primary cultured hippocampal neurons suggest that D1/D5 dopamine receptor activation by SKF38393 induces a decrease in the co-localisation of α-synuclein with bassoon. This points to changes in the presynaptic apparatus and potentially altered neurotransmitter release properties under these conditions.

### Behavioural analysis

The above proteomic (*cf*. Additional file 1: Table S1), immunoblot (*cf*. Figure 3), and immunocytochemical analyses (*cf*. Figure 4) indicate that the abundance and/or subcellular localisation of α-synuclein are subject to dopaminergic regulation. This protein has been implicated in the modulation of glutamatergic and dopaminergic mechanisms of neurotransmission [30,31], which were also shown to be of critical importance for FM discrimination learning and memory in rodents [21,22,35]. To assess the role of α-synuclein for FM discrimination learning and its dopaminergic modulation, we used mice of three C57BL/6J substrains. C57BL/6JOlaHsd mice (here referred to as subpopulation 6JOla) harbour a spontaneous chromosomal deletion of α-synuclein and multimerin-1 loci [36], which is associated with alterations in dopamine neurotransmission [37,38], while C57BL/6JCrl and C57BL/6JRccHsd mice constitute a subpopulation (referred to as 6J) with no such deletion.

Mice were trained daily for 16 days to discriminate between rising and falling FMs. Learning behaviour and performance were studied in a GO/NO-GO task aiming at avoidance of a mild foot-shock by crossing the hurdle in a two-way shuttle box. The discrimination performance was quantified by the discrimination rate *D*, that is, the difference between the relative frequencies of CR+ and CR− (*i.e*., hurdle crossings in response to the reinforced and unreinforced stimuli, respectively) per training session. During pilot studies performed to adapt this paradigm for mice, we observed notable tendencies of enhanced learning and reduced asymptotic performance in 6JOla mice as compared to 6J mice (data not shown). Combined with previous results [15,21,22], we hypothesised that dopamine signalling during early stages of differential conditioning to FMs determines the efficiency of subsequent learning and memory formation and asymptotic behavioural performance. Consequently, we examined effects of D1/D5 dopamine receptor selective antagonists and agonists intraperitoneally applied immediately after the first session of conditioning to FMs on differences in *D* between the 6JOla and 6J subpopulations.

Data from the two substrains of the 6J subpopulation, C57BL/6JCrl and C57BL/6JRccHsd, were combined due to their equal discrimination performance (see Additional file 4: Table S3). During the course of training, all experimental groups showed significant increases in the relative frequencies of CR+ over the values of CR−, indicating that both the 6JOla and the 6J subpopulation were able to learn the task under each of the pharmacological treatment conditions. Hurdle crossings during the intertrial intervals did not significantly differ between subpopulations, implying similar levels of arousal and general activity (see Additional file 4: Tables S4-S9 for the relative frequencies of CR+ and CR− and the numbers of intertrial crossings).

Analysis of the discrimination rates *D* with a 6 × 2 × 16 (pharmacological treatment × subpopulation × training session) repeated-measures ANOVA, with session as the repeated measure, revealed a significant main effect of session (*F*15,1350 = 155.423, *P* < 0.0001) and significant treatment x subpopulation (*F*5,90 = 2.487, *P* = 0.0371), session × treatment (*F*75,1350 = 1.709, *P* = 0.0002), session × subpopulation (*F*15,1350 = 14.609, *P* < 0.0001), and session × treatment x subpopulation (*F*75,1350 = 1.868, *P* < 0.0001) interactions.

**Figure 5:**
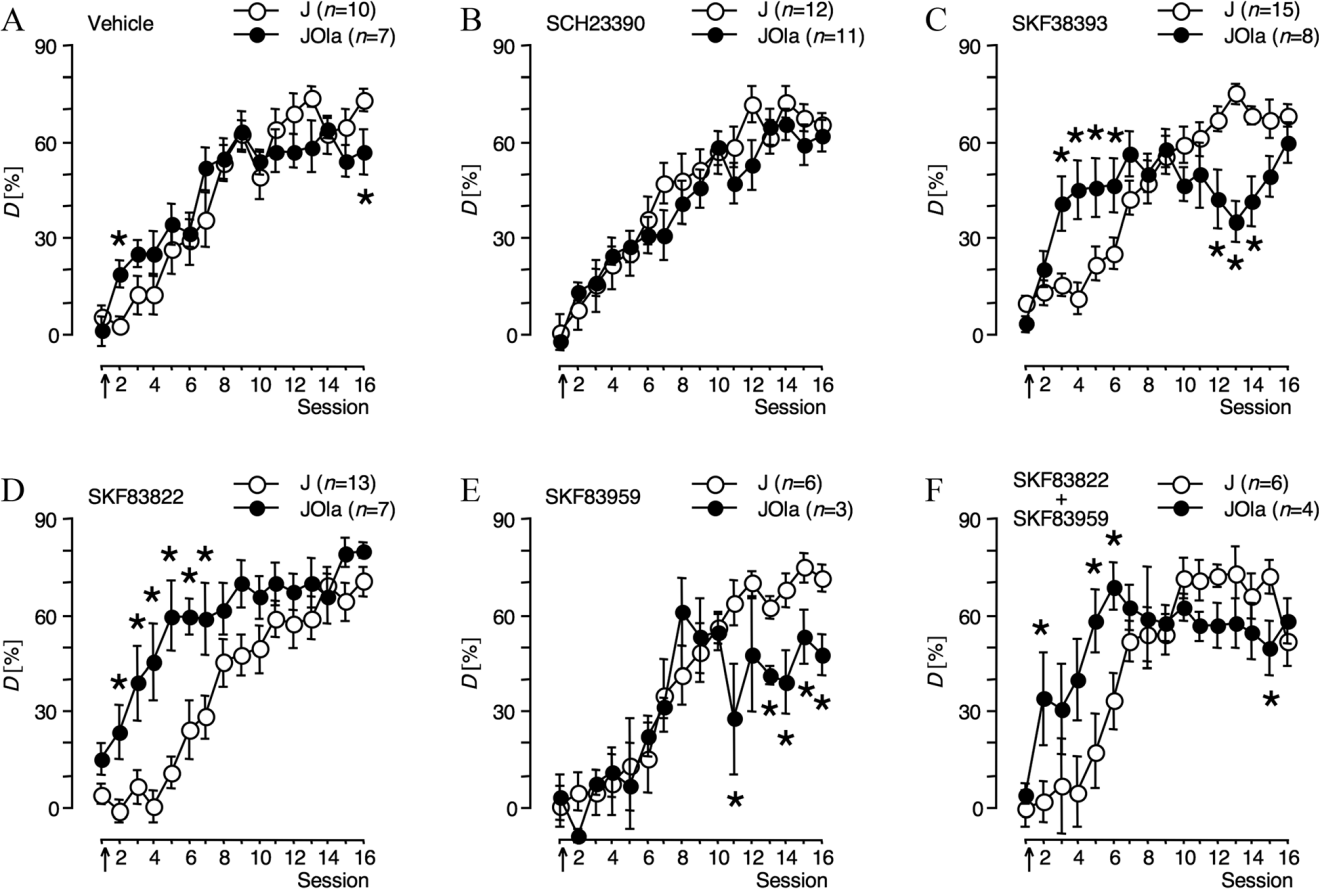
**D1/D5 dopamine receptor-selective drugs differentially modulate frequency-modulated tone discrimination learning of 6JOla and 6J mice.** Mice were trained in daily sessions to discriminate between rising and falling frequency-modulated tones. After session 1, they received intraperitoneal injections of vehicle **(A)**, SCH23390 **(B)**, SKF38393 **(C)**, SKF83822 **(D)**, SKF83959 **(E)**, or a combination of SKF83822 and SKF83959 **(F)**. Arrows indicate the approximate injection time. Discrimination rates *D* are presented as means ± S.E.M. *n* refers to the number of mice. ANOVA values are described in the text. *P < 0.05, significantly different from the corresponding value of the 6J subpopulation (unpaired *t*-test).

To assess the origins of these interactions, *D* was analysed separately within training sessions, subpopulations, and treatment conditions. Within-session 6 × 2 (pharmacological treatment × subpopulation) ANOVA revealed significant main effects and/or interactions of treatment condition and subpopulation in sessions 2–7 and 11–16, but not in session 1 (Additional file 4: Table S10), implying comparable acquisition performance during initial training. Within-subpopulation 6 × 16 (pharmacological treatment x training session) repeated-measures ANOVA indicated that pharmacological treatments significantly affected FM discrimination of 6JOla (main effect of treatment: *F*5,34 = 2.547, *P* = 0.0463; session × treatment interaction: *F*75,510 = 2.623, *P* < 0.0001) but not of 6J mice (main effect of treatment: *F*5,56 = 0.545, *P* = 0.7411; session × treatment interaction: *F*75,840 = 1.095, *P* = 0.2787). Interestingly, 2 × 16 (subpopulation × training session) repeated-measures ANOVA within the pharmacological treatment conditions uncovered differential effects of dopaminergic treatments on the learning curve slopes in the two subpopulations (Figure 5). Vehicle-treated 6JOla mice (Figure 5A) initially learned faster, but reached lower final performance levels than corresponding 6J mice (session × subpopulation interaction: *F*15,225 = 2.884, *P* = 0.0004). The D1/D5 dopamine receptor antagonist SCH23390 (Figure 5B) abolished (main effect of subpopulation: *F*1,21 = 0.518, *P* = 0.4797; session × subpopulation interaction: *F*15,315 = 1.337, *P* = 0.1784), whereas the agonist SKF38393 (Figure 5C) augmented (session × subpopulation interaction: *F*15,315 = 10.362, *P* < 0.0001) the differences between subpopulations in ascending and asymptotic curve regions. SCH23390 and SKF38393 were reported to interfere with both of the known signalling modes of D1/D5 receptors, *i.e.*, ADCY- and PLC-mediated pathways [25]. However, differential effects of SKF38393 and SKF83959 on α-synuclein abundance (compare Additional file 1: Table S1) might point to differing roles in ADCY- and PLC-mediated signalling. To assess impacts of post-learning activation of each pathway on subsequent FM discrimination learning and performance, the D1/D5 receptor agonists SKF83822 and SKF83959 were used. SKF83822 (Figure 5D), reported to stimulate ADCY-linked and to desensitise PLC-linked D1/D5 signalling [25,39], accentuated differences between subpopulations in the ascending section of the learning curve but abolished those near the asymptotic section (main effect of subpopulation: *F*1,18 = 9.349, *P* = 0.0068; session × subpopulation interaction: *F*15,270 = 3.299, *P* < 0.0001). Conversely, SKF83959 (Figure 5E), reported to antagonise dopamine-mediated stimulation of ADCY and to preferentially stimulate PLC-linked signalling [26,27], abolished differences between subpopulations at ascending but accentuated those near asymptotic learning curve sections (session × subpopulation interaction: *F*15,105 = 2.188, *P* = 0.0110). Notably, effects of SKF83822 and SKF83959 co-injection (Figure 5F; session × subpopulation interaction: *F*15,120 = 4.258, *P* < 0.0001) resembled those of SKF38393 (Figure 5C).

Thus, altered dopamine neurotransmission in α-synuclein-deficient mice revealed that distinct D1/D5 dopamine receptor signalling modes may control different aspects of memory consolidation.

## Discussion

When applied locally to the AC of gerbils, the D1/D5 dopamine receptor agonists SKF38393 and SKF83959 induced differential changes in 2D gel electrophoretic protein profiles of the AC itself and of frontal-cortical, hippocampal, and basal brain regions. SKF38393, reported to stimulate ADCY- and PLC-linked D1/D5 signalling, induced the most pronounced changes in the AC and the ST, the fewest in the FC. In contrast, changes induced by SKF83959, reported to preferentially stimulate PLC, were most pronounced in the FC. Regulated protein spots included, among others, proteins of mitochondria and energy metabolism, cytoskeleton and scaffolding components, and proteins putatively involved in inter- and intracellular communication. Abundance and/or subcellular localisation of α-synuclein also seem to be subject to dopaminergic regulation. Studies on α-synuclein-deficient mice revealed that systemic pharmacological interference with ADCY- and PLC-mediated D1/D5 dopamine receptor signalling modes after the initial session of auditory discrimination training preferentially affected acquisition rates over subsequent learning episodes and asymptotic memory performance, respectively.

### General considerations

Memory-supporting synaptic plasticity phenomena are assumed to depend on the combined action of different mechanisms, including mechanisms that create the potential for a lasting change in synaptic efficacy [6]. Learning an olfactory discrimination task, for example, has been shown to induce an enhancement of learning capability accompanied by a series of physiological and morphological modifications in cortical neurons (for review, see [40,41]). In particular, intrinsic neuronal excitability is increased, synaptic transmission is enhanced, and the number of dendritic spines is increased. Such long-lasting activity-dependent changes were shown to correlate with learning abilities across species and tasks, require neuromodulatory actions, such as D1/D5 dopamine receptor activity, and depend on protein synthesis and cytoskeletal reorganisation [42-46]. As such learning-related modifications are spread throughout the neuronal ensemble, and as they disappear without memory loss, they probably are not mechanisms by which specific memories are stored. Rather, they may enable relevant neuronal ensembles to enter a ‘learning mode’ for a time window in which activity-dependent synaptic modifications are more likely to occur [41]. Accordingly, cortical map plasticity was recently shown to improve auditory discrimination learning – presumably by increasing the number of responsive circuits in multiple brain regions [7].

Results of our previous studies on FM discrimination are consistent with this view: During and shortly after learning of this task, cortical dopamine release is increased, and translation-dependent changes with different kinetics and behavioural consequences are induced in the AC. Whereas one process enables storage and retrievability of the newly acquired memory until the next training session performed 1 day later, another process, downstream of D1/D5 dopamine receptors and mTOR, enhances the predisposition for learning and memory formation on subsequent training days [17,18,21,22]. Adaptive synaptic proteome changes recently monitored in cortical, hippocampal, and striatal regions of mice after FM discrimination learning [19] may be associated with both permissive and memory-stabilising processes. The present findings of molecular alterations in analogue brain regions of naïve gerbils after artificial activation of auditory-cortical D1/D5 dopamine receptors are assumed to preferentially reflect, at least in part, learning-induced permissive mechanisms.

### Proteome changes: regional aspects

In the current study, local injection of benzazepine D1/D5 dopamine receptor agonists into the gerbil AC induced proteome changes not only in the AC itself, that is, the target region of injection, but also in distant brain regions such as the frontal cortex, hippocampus, and striatum. As demonstrated in the rat, clearance of benzazepines after intracerebral injection occurs within 1–3 h, and the extent of diffusion hardly exceeds 1.5 mm [47,48]. Supposing similar penetration kinetics in the gerbil AC with a rostrocaudal extent of roughly 3 mm [49], this implies that the behavioural effects of locally applied benzazepines on FM discrimination memory [21,22] and the molecular effects monitored in different brain regions in the present study were induced by pharmacological actions shortly after drug injection in the vicinity of the infusion sites in the AC.

In the gerbil, tracer studies have discovered numerous connections of the AC to cortical and subcortical structures [20]. FM discrimination involves an instrumental conditioning in which the behavioural meaning, GO or NO-GO, of two sounds is determined from the probability to avoid punishment by mild foot-shock. Thereby, AC neurons not only distinguish the learnt meaning of the sounds but also seem to be involved in the selection of behavioural response strategies and the appraisal of reward feedback. Anatomical substrates of such non-auditory and cognitive influences include feedback connections to the AC from multiple brain areas [15,20]. FM discrimination presumably involves an interaction, among others, between the AC and the FC. This has been concluded from microdialysis studies on auditory- and prefrontal-cortical regions showing elevated dopamine responses during and shortly after shuttle box avoidance conditioning to FMs [23,24,50]. Prefrontal dopamine flux and auditory-cortical activity have been shown to influence cognitive functions, such as attention and working memory [48,51], and downstream dopaminergic targets, in particular the striatum [52-54]. Dopaminergic modulation in prefrontal and striatal substructures is critical for the timing of processes assumed to support acquisition and performance of instrumental actions [55]. In the present study, proteome changes after activation of auditory-cortical dopamine receptors were monitored in frontal-cortical, hippocampal, and striatal brain regions. This might reflect plastic alterations that facilitate the interaction of these structures, which are implicated in, among others, novelty computation, salience attribution, and reward processing during learning and memory formation. This is consistent with models whereby dopamine signals in the midbrain-hippocampus loop and in frontostriatal circuitry regulate consolidation and strengthening of long-term memory [56,57].

Our previous studies suggest that auditory-cortical D1/D5 dopamine receptor activation mainly acts on processes relevant for consolidation of the newly acquired FM discrimination into long-term memory [21,22]. Mechanisms of memory consolidation are assumed to involve interactions of networks in multiple brain regions over days or weeks [58,59]. The HC is believed to integrate information from distributed cortical networks into a coherent memory trace and to mediate its temporary storage and retrieval. To enable remote memory storage and retrieval, neurons in the cortex, in particular in frontal regions, must undergo a tagging process early upon encoding [5]. Consolidation of the memory trace at the cortical level would then occur slowly via repeated reactivation of hippocampocortical networks. In the light of these considerations, regional differences in proteome changes observed in the present study 24 h after intra-AC injection of SKF38393 and SKF83959 (Figure 1) may be indicative of agonist effects on different aspects of memory consolidation. SKF38393-induced proteome changes in the HC considerably exceeded those in the FC. Supposed that in the FM discrimination paradigm the HC plays a temporary role in the management and consolidation of the newly acquired memory and that the observed alterations support these mechanisms, the present findings provide a plausible explanation for the dynamics of the facilitating effect of SKF38393 on FM discrimination memory, which is detectable already 1–2 days after its auditory-cortical infusion [21]. In contrast, 24 h after SKF83959 infusion into the AC, the most prominent changes were detected in the FC. Supposed that in the FM discrimination paradigm neurons in the FC must undergo a tagging process during encoding to enable, over time, storage and retrieval of the remote memory and that the observed alterations support these mechanisms, the present findings may explain a delay of several days in behavioural effects that we have observed in this paradigm when SKF83959 was locally applied to the gerbil AC (data not shown). Thus, differential proteome changes in multiple brain regions and the dynamics of behavioural effects after injection of SKF38393 and SKF83959 into the AC may be indicative of impacts of distinct D1/D5 dopamine receptor signalling modes on different aspects of memory consolidation.

### Proteome changes: molecular and cellular aspects

In the present study, two fractions were prepared from brain regions and examined for changes in their protein composition. The TP fraction includes integral membrane proteins, membrane-associated proteins, and cytosolic proteins. The SP fraction includes cytoskeletal and synaptic scaffolding proteins, membrane proteins and signalling components that are tightly associated with the synaptic cytomatrices, and extracellular matrix proteins [60]. These proteins show a surprisingly high rate of turnover or mobility [61-63]. They are dynamically regulated by protein modifications, which may decide about dynamic redistribution, proteasome-dependent degradation or local synthesis of specific proteins. Thus, the abundance of a protein in the TP and SP fractions may depend on either its degradation and synthesis rates during plasticity processes or the strengths of protein–protein interactions that can be regulated by posttranslational modification.

Mitochondrial proteins and proteins implicated in energy metabolism were among those proteins that were most frequently recognised in regulated spots from the analysed protein fractions and brain regions after both SKF38393 and SKF83959 treatment (Additional file 1: Table S1; Figure 2). Mitochondria are highly mobile organelles. In neurons, they are recognised as not only a power station, but also as a signalling platform involved in fundamental events in the formation and plasticity of neuronal circuits (for review, see [64]). They move within and between subcellular compartments involved in neuroplasticity (synaptic terminals, dendrites, cell body and the axon). By generating energy and regulating subcellular calcium homoeostasis, mitochondria may play important roles in controlling fundamental processes in neuroplasticity, such as transport of various RNA and protein cargos, membrane turnover, and cytoskeletal dynamics.

Proteins of the category “cytoskeleton, scaffolding, extracellular matrix” also count among those most frequently identified in regulated spots in the present study, irrespective of the used agonist and analysed brain region. In conjunction with the regulation of components of proteasome-mediated protein degradation, these findings suggest that structural elements, including the subsynaptic cytoskeleton, are highly modulated in various brain regions after induction of cortical dopaminergic activity, which, in turn, might support learning-induced plastic rearrangements. Indeed, cytoskeletal proteins were shown to be down-regulated 6 h following the induction of long-term potentiation [65], a well-characterised cellular form of synaptic plasticity thought to underlie learning and memory. Moreover, studies on the rat HC suggest that learning-induced processes may modulate the monomer/polymer balance of major components of the cytoskeleton for up to at least 24 h [66]. Accordingly, our recent studies on mice demonstrated the reduced abundance of giant multi-domain proteins in the synaptic junctional protein fraction derived from cortical and subcortical brain regions after FM discrimination training [19]. In view of the critical importance of a majority of these proteins for the organisation of components of the cellular structure, that is, the cytoskeleton, scaffolds, and the extracellular matrix, these changes might reflect, in part, learning-induced reductions in the association of proteins with subsynaptic cytomatrices in support of synaptic remodelling during future learning events and/or memory consolidation processes at the systems level.

As previously shown in the FM discrimination paradigm [21], the induction of a consolidation-enhancing trace by SKF38393 requires mTOR activity in the AC. Among the cellular functions of mTOR is the translational control of distinct classes of mRNAs, including transcripts that encode constituents and regulators of the translational machinery. Therefore, one possible explanation of the memory-enhancing agonist effect was that D1/D5 dopamine receptor activation might induce an increase in the translational capacity of neurons and/or neuronal compartments in the AC, which, in turn, might enhance – for hours or even days – the ability to synthesise plasticity-related proteins locally on demand. In the present study, we found a differential regulation of several RNA-binding proteins after agonist treatments (Additional file 1: Table S1; Figure 3). This includes, for example, hnRNPs K, L, and D (alias AUF1), which have been implicated in the regulation of mRNA stability and translation in mammalian cells [67,68], and hnRNP A2, which is part of an mRNA trafficking system in neural cells that is involved in the targeting of certain mRNAs to dendrites [69]. Induction of synaptic plasticity was shown to increase the abundance of various hnRNPs at synapses, where they may control, among others, the expression of cytoskeletal components, spine formation, and synaptic transmission and plasticity [70-73]. Accordingly, we found differential regulation of hnRNPs by SKF38393 predominantly in the synaptic junctional protein-enriched fraction. However, contrary to our expectations, hnRNPs were recognised almost exclusively in down-regulated spots from the AC, but instead in up-regulated spots from the HC. Supposed that the abundance of these proteins in the SP fraction reflects the translational capacity in dendritic and/or synaptic compartments, these findings imply that, when infused into the AC, SKF38393 does not induce a longer lasting activation of the local translational machinery in the AC, but in the HC. The results can be explained by postulating that D1/D5 receptor activation by SKF38393 in the AC induces rapid and transient mTOR-mediated, translation-dependent alterations in auditory-cortical signal transduction, which, in turn, causes an increase of the local translational capacity in associated memory-related brain regions, such as the HC. Activation of previously silent synapses by dopaminergic stimulation [74], or dopaminergic regulation of dendritic protein synthesis by miniature synaptic events [75,76] would be only two conceivable mechanisms of the postulated mTOR-mediated changes in auditory-cortical signalling. Analogous mechanisms, but probably via other downstream effectors, might mediate the effects of SKF83959, which, when applied to the AC, mainly induced an up-regulation of RNA-binding proteins in the FC.

### α-Synuclein and dopaminergic modulation of learning and memory

The predominantly presynaptic protein α-synuclein, associated with several neurodegenerative diseases, is implicated, among others, in the regulation of different aspects of neurotransmission (for review, see [77,78]). α-Synuclein was shown to be involved in almost all processes related to dopamine synthesis and release. It is an essential presynaptic, activity-dependent negative regulator of dopamine neurotransmission. The release of some other neurotransmitters, such as glutamate and norepinephrine, was also shown to be regulated by α-synuclein.

Results collected in *in vivo* and *in vitro* experiments of the present study imply an SKF38393-induced increase in the abundance of α-synuclein and decrease in its co-localisation with bassoon, a presynaptic cytomatrix protein used as marker protein of the active zone of neurotransmitter release [34,79]. Both α-synuclein and bassoon are implicated in presynaptic mechanisms of neurotransmission [30-32,80-83]. D1/D5 dopamine receptor-induced changes in their localisation might, therefore, be reflective of dopaminergic effects on the release of neurotransmitters, such as dopamine and glutamate, which, in turn, might impact learning and memory formation. In addition, α-synuclein was shown to be of critical importance for processes in other cellular compartments, such as mechanisms of endoplasmic reticulum-to-Golgi transport or the endocytosis of NMDA-type glutamate receptors [84,85].

Experimental evidence suggests a role of α-synuclein for several forms of long-lasting synaptic plasticity, for example, in hippocampal and corticostriatal signalling, and for song learning in birds [78]. Accordingly, improved spatial learning and memory in rats has recently been shown to correlate with hippocampal immunoreactivity to α-synuclein [86]. To assess the role of α-synuclein for FM discrimination learning and its dopaminergic modulation, we used different subpopulations of C57BL/6J mice. The 6JOla subpopulation differs from their 6J ancestors in possessing a chromosomal deletion resulting in the loss of two genes encoding α-synuclein and multimerin-1, which is associated with alterations in dopamine neurotransmission [37,38,87]. The expression of multimerin-1 has not been observed in brain [36], implying that any behavioural phenotype of 6JOla mice is not confounded by the lack of this protein. 6JOla mice have a normal life expectancy, show no obvious major sensorimotor or motivational deficits, and are able to learn spatial and olfactory tasks normally [88,89].

In the FM discrimination paradigm, 6JOla mice initially learned faster but reached lower final performance levels than the corresponding 6J mice (Figure 5). The higher accuracy of 6JOla mice during the first days of training was mainly due to a lower relative frequency of CR− (*cf*. Additional file 4: Tables S4-S9). This could point to a recently suggested role for α-synuclein in ‘waiting’ impulsivity [90]. As a consequence, 6JOla mice might have shown less spontaneous hurdle crossings in the shuttle box and, thus, reached higher discrimination accuracy than 6J mice. However, hurdle crossings during the intertrial intervals did not significantly differ between subpopulations, and the difference in CR− disappeared during the following days of training, suggesting that the mutation did not cause differences in general activity, arousal, and impulsivity that might have interfered with the discrimination performance. On the other hand, 6JOla mice were shown to have a greatly increased rate of operant behaviour during intracranial self-stimulation [91], which was explained by a sensitised brain reward system due to a lack of the negative impact of α-synuclein on dopaminergic neurotransmission. Indeed, the differences in the learning curves of 6JOla and 6J mice in the present study were completely abolished by the D1/D5 dopamine receptor antagonist SCH23390 but substantially intensified by the D1/D5 agonist SKF38393 when systemically applied immediately after the initial training session. Moreover, preferential activation of distinct downstream effectors of D1/D5 dopamine receptors by effector-selective agonists had differential impacts on the performance differences at the ascending and asymptotic parts of the learning curve. Together, these findings suggest that α-synuclein is involved in dopamine signalling modes via D1/D5 receptors that impact different mechanisms relevant for learning and memory formation.

## Conclusions

Proteomic findings of the present study suggest that activation of distinct D1/D5 dopamine receptor signalling modes in the auditory cortex may induce differential changes of protein profiles in associated brain structures, which may be supportive for structural and functional plastic rearrangements during future events of learning and memory consolidation. Consistent with this view, behavioural results on the discrimination of complex sounds suggest that distinct dopamine signalling modes via D1/D5 receptors impact different mechanisms relevant for learning and memory formation. Both modes are activated during initial encoding. Whereas ADCY-linked signalling may facilitate acquisition during subsequent learning episodes and/or mechanisms that ensure storage and retrieval of newly acquired memories, PLC-linked signalling may control mechanisms determining the formation of enduring memories. The sensitivity of these mechanisms to pharmacological dopaminergic interference was demonstrated in α-synuclein-deficient mice, implying a role of this neuronally expressed protein in D1/D5 dopamine receptor signalling.

## Methods

### Animals

Male 3-month-old Mongolian gerbils (*Meriones unguiculatus*) were used for proteomic studies. For behavioural experiments, male 3-month-old C57BL/6J mice of the substrains C57BL/6JOlaHsd (Harlan), C57BL/6JCrl (Charles River) and C57BL/6JRccHsd (Harlan) were used. The animals were housed at most in groups of five and given free access to standard laboratory chow and tap water on a 12-h light/dark cycle (light on at 6 a.m.). All animal procedures were conducted during the light period. Animal experimentation was approved by the animal care committee of the Land Sachsen-Anhalt in accordance with the regulations of the German Federal Law on the Care and Use of Laboratory Animals and with NIH guidelines.

### Pharmacological agents

The D1/D5 dopamine receptor-selective agonists SKF38393 (Sigma), SKF83822 (Tocris) and SKF83959 (Sigma), and the D1/D5 dopamine receptor-selective antagonist SCH23390 (Sigma) were dissolved in 0.9% saline and adjusted to ≈ pH 7. The doses used – that is, 0.06 μg/μL = 0.2 mM SKF38393 and 0.5 μg/μL = 1.25 mM SKF83959 for intracortical injections, and 5 mg/kg SKF38393, 0.625 mg/kg SKF83822, 2.5 mg/kg SKF83959, and 0.1 mg/kg SCH23390 for intraperitoneal injections – were based on our previous studies on mechanisms of FM discrimination memory in the gerbil auditory cortex [21,22] in conjunction with studies on the pharmacological efficacy of the agonists [27].

### Surgical procedures, intracortical injections, and brain dissection

Surgery and intracortical injections were performed bilaterally as described in detail elsewhere [17,92]. In brief, on the day before intracortical injections, gerbils were deeply anaesthetised (4 mg ketamine and 3 mg xylacine per 100 g body weight), the cranial skin was disinfected and incised, and 3 holes of about 1 mm in diameter were drilled per hemisphere into the skull at locations covering the primary, anterior, and posterior fields of the AC. After surgery, gerbils were allowed to recover for 1 day before 1-μL portions of drug solution or vehicle (0.9% saline) were applied per target region under light halothane anaesthesia over a period of 4 min. Injections were repeated after an interval of 2 h. The procedure of positioning the injection tracks has previously been validated [17]. Twenty-four hours after the first injection, gerbils were killed by decapitation, the AC, FC, HC, and ST were localised on the basis of their stereotactic coordinates, surgically removed, frozen in liquid nitrogen, and stored at −80°C as described [19].

### Tissue fractionation

TP and SP fractions were prepared from the frozen tissue samples as described [60]. Briefly, brain tissue was homogenised in 300 μL of homogenisation buffer, containing 5 mM Tris/HCl, pH 8.1, 0.5% Triton X-100, complete mini PI protease inhibitor cocktail (Roche), halt protease and phosphatase inhibitor cocktail (Thermo Scientific), 1 mM sodium molybdate (Roth), 2 mM imidazole (Sigma), 4 mM sodium tartrate dihydrate (Roth), and 10 μM cantharidin (Roth). After incubation for 1 h at 4°C, samples were centrifuged at 100,000 g for 1 h. The resulting pellets were re-homogenised in 300 μL of homogenisation buffer and centrifuged again. The combined supernatants represented the TP fraction; the final pellets, resuspended in 200 μL of homogenisation buffer, represented the SP fraction. Proteins of both fractions were precipitated with acetone, washed, and lyophilised. Before further use, protein concentrations of redissolved samples were measured using a Bradford assay (BioRad). The suitability of the fractionation method for gerbil brain tissue has been confirmed by immunoblot analysis (Additional file 5: Figure S2).

### Proteome analysis

#### 2D Gel electrophoresis

TP and SP fractions from individual animal brain regions were analysed. 2D gel electrophoresis was performed as described [93,94]. IPG-gel strips (18 cm; pH 3–11 NL; Amersham) were incubated in 400 μl of IPG buffer (Amersham) containing 700 μg of protein from individual brain regions for analytical purposes or 2 mg of protein from pooled brain tissue for preparative purposes. Isoelectric focusing was performed using the Ettan-IPGphor system (Amersham). Separation in the second dimension was performed on 11% polyacrylamide gels using the EttanDALT system (Amersham). Proteins in the gels were visualised by silver staining [95] for quantitative analysis and by Coomassie Blue G-250 [96] for mass spectrometric analysis. Image analysis software, PDQuest v8.1 (BioRad), was used to compare the digitised (GS-800, BioRad) images and detect protein spots whose optical densities were significantly (see below) increased or decreased after pharmacological treatment compared to control. Protein spots with similar optical density values as in control animals (from factor 0.9 to 1/0.9) were excluded from further analyses. Replicate 2D gels could not be run due to the limited protein amount extractable from individual animal brain regions.

#### Mass spectrometry

To identify proteins in differentially regulated 2D gel spots, nano-LC-ESI-iontrap mass spectrometry was used as described [89]. Protein spots of interest were manually excised from Coomassie stained preparative gels, washed and de-stained two times with vigorous shaking using 25 mM ammonium bicarbonate/50% (v/v) acetonitrile (ACN), followed by ACN. Digestion was performed by trypsin (Promega) added onto the dried gel pieces and incubation overnight at 37°C. Peptides were extracted with 50% ACN/0.1% (v/v) trifluoroacetic acid (TFA), sonicated, and dried. Peptides were resuspended in 1% TFA, purified with reversed-phase C18 ZipTip nano-columns (Millipore), eluted with 0.1% TFA/70% ACN, and dried. For mass spectrometric analysis, samples were redissolved in 10 μl of 2% ACN/0.05% TFA and subjected to Ultimate/Swichos Nano-HPLC (Dionex). The nano-HPLC was coupled online via a nano-spray source (Bruker) to an Esquire HCT Iontrap mass spectrometer (Bruker). Mass spectra were acquired in positive MS/MS-mode, tuned for tryptic peptides, processed using Data Analysis and BioTools software packages (Bruker), and catalogued with ProteinScape (Bruker Daltonics). The used mass spectrometric approach did not produce data sufficient for spectra counting. With respect to tune the measurements for maximal sequence coverage we used active precursor exclusion after the first MS/MS run. An already measured precursor was not selected again for a MS/MS experiment within a retention time window of 1 min. The average peak width at half maximum was 15 sec. For the peptide sequence matching, Mascot (Matrix Science) was used. Database search and functional annotation for identified proteins using UniProt (www.uniprot.org) and SynProt (http://www.synprot.de; [29]) were taxonomically expanded to mammals due to the very low numbers of gerbil entries. Annotation performed with rodent proteins yielded comparable results (*cf.* Figure 2 and Additional file 6: Figure S3).

### Immunoblot analysis

For Western blot analysis, equal amounts of protein (adjusted from Coomassie stains) were separated by SDS-PAGE and transferred to a nitrocellulose membrane (Whatman). After blocking with 5% (w/v) low-fat milk in TBS/0,1%/Tween-20 (Roth), quantitative immunoblot analyses were performed using primary antibodies against hnRNP K (polyclonal, anti-rabbit, 1:500, Abcam), hnRNP L (monoclonal, anti-mouse, 1:2000, Abcam), α-synuclein (monoclonal, anti-mouse, 1:1000, BD Biosciences), NEFL (monoclonal, anti-rabbit, 1:50000, Novus), and β-actin (monoclonal, anti-mouse, 1:4000, Sigma), followed by appropriate HRP-coupled secondary antibodies in 5% low-fat milk/TBS/0,1% Tween-20. Immunoreactive bands were detected using HRP-chemiluminescence substrate (Pierce ECL Western Blotting Substrate, Thermo Scientific) and exposition on Amersham Hyperfilm (GE Healthcare). Signals on the developed films were digitised (GS-800, BioRad) and quantified using QuantityOne v4.6.9 (BioRad). Signals were normalised to Coomassie stains because the present proteome analyses have revealed that the abundance of proteins commonly used for normalisation (e.g., GAPDH and cytoskeletal components, such as tubulin and actin isoforms) also showed changes in response to drug treatment (*cf*. Additional file 1: Table S1).

### Immunocytochemical analysis

Four independent experiments were performed sequentially. Primary cultures of hippocampal neurons were prepared from E18 Wistar rats using 0.1% (w/v) trypsin (Gibco) and 0.01% (w/v) DNase (Roche), plated at 20,000 cells/cm2 onto poly-D-lysine (Sigma) coated coverslips, and maintained in neurobasal medium containing B-27, L-glutamine, and antibiotics (Gibco) at 37°C in 5% CO2/95% H2O saturated air as described [97]. For co-localisation analysis, DIV21 neurons were incubated for 3 h with replaced medium containing either no SKF38393 (control) or 100 μM SKF38393, fixed in 4% (w/v) paraformaldehyde/PBS for 7 min, quenched with 25 mM glycine/PBS, pH 7.4/0.5 mM calcium chloride/0.5 mM magnesium chloride (Roth) for 30 min, and blocked and permeabilised in 0.2 mg/ml saponin/5% (w/v) bovine serum albumin (Invitrogen)/10% (v/v) horse serum (Invitrogen)/PBS, pH 7.4, for 1 h at room temperature. Immunocytochemical staining was performed using primary antibodies against bassoon (polyclonal, anti-rabbit, 1:1500, kindly provided by Dr. Wilko Altrock, Leibniz Institute for Neurobiology, Magdeburg, Germany), α-synuclein (polyclonal, anti-guinea pig, 1:1500, Abcam) and PSD95 (monoclonal, anti-mouse, 1:500, NeuroMab) overnight at 4°C, followed by the appropriate secondary antibody (Alexa-488 anti-rabbit IgG, 1:1000, Invitrogen; cy3 anti-guinea pig IgG, 1:1000, Dianova; cy5 anti-mouse IgG, 1:1000, Dianova) for 1 h at room temperature. Co-localisation of immunofluorescence signals was assessed in dendrites of 10 neurons per experiment and treatment condition. Images (2048 × 2048 Pixel; 12 bit; 63× objective; 1,5× zoom; 3× averaging; 0,13 μm stacks; 400 Hz) were acquired using a TCS SP5 microscope (Leica), argon laser excitation of Alexa-488 (emission wavelength 519 nm), DPSS 561 laser excitation of Cy3 (emission wavelength 570 nm), and helium-neon laser excitation of Cy5 (emission wavelength 670 nm). Exposure times were kept identical between treatment groups and below grayscale saturation. Images of dendrites were straightened (ImageJ, NIH) and deconvolved (AutoQuant X2, MediaCybernetics) using the following parameters for 3D deconvolution: deconvolution method, adaptive blind algorithm; 15 iterations. Co-localisation analysis was assessed using Imaris v6.4.2 (Bitplane Scientific Solutions) based on the method of Costes et al. [98]. Co-localisation was defined as the overlap of two (bassoon and α-synuclein) or three (bassoon and α-synuclein versus PSD95) channels in three dimensions and calculated automatically by the program. The degree of co-localisation was represented by “percentage of material co-localised” and the Pearson correlation coefficient in voxels with co-localisation above the threshold. Dendrites from ten neurons from four independent experiments were analysed for each treatment.

### Behavioural analysis

Mice were trained once per day, for 16 sessions in total, on a foot-shock reinforced shuttle box avoidance GO/NO-GO procedure to discriminate the directions of modulation of FMs [19]. Before each training session, mice were allowed to habituate for 3 min to the training chamber without acoustical stimulation and foot-shock. During sessions, animals were trained to discriminate between conditioned stimuli (CSs) consisting of sequences (250-ms tone, 250-ms pause) of an ascending (4–8 kHz, CS+) and a descending FM (8–4 kHz, CS−). A training session consisted of 60 trials, that is, 30 presentations of each CS+ and CS− in a pseudo-randomized order, and lasted ≈ 25 min. The mean intertrial interval was 15 s. To avoid mild electrical foot-shock, mice had to cross the hurdle of the shuttle box within 6 s of CS+ presentation and to suppress this response within 6 s of CS− presentation. Hurdle crossings within 6 s upon the onset of CS+ and CS− were regarded as correct conditioned responses (CR+) and false alarms (CR−), respectively. For each session, the numbers of CR+ and CR− were monitored and the relative frequencies of CR+ and CR− were calculated as percentage of trials with presentations of CS+ and CS−, respectively. To assess general arousal and activity of the experimental animals, the intertrial activity, that is, the numbers of hurdle crossings occurring between the trials of each session, were monitored. To quantify the discrimination performance, the discrimination rate *D*, that is, the difference between the relative frequencies of CR+ and CR−, was calculated. Pharmacological treatments were applied immediately after completion of the first training session. Six sets of experiments with different treatment conditions were performed. In each set, C57BL/6JOlaHsd, C57BL/6JCrl and C57BL/6JRccHsd mice were trained in parallel and received identical intraperitoneal injections either of vehicle (0.9% saline), SCH23390, SKF38393, SKF83822, SKF83959, or of a combination of SKF83822 and SKF83959.

### Statistics

Protein-analytical data collected by proteome or immunoblot analyses were evaluated using Mann-Whitney’s *U*-test. For evaluation of immunocytochemical and behavioural data, one- and two-way ANOVA and repeated measures ANOVA (with training session serving as the repeated measure) were performed as indicated, using StatView 5.0.1 (SAS). Individual data points were compared using Student’s two-sided *t*-test for paired or unpaired comparisons, as indicated. *P* values of <0.05 were considered as statistically significant.

## Additional files

Additional file 1: Table S1. Overview of proteins identified in differentially regulated spots. Additional file 2: Table S2. Proteins identified in differentially regulated spots. Additional file 3: Figure S1. Confirmation of co-localisation analysis. Additional file 4: Tables S3-S10. Behavioural data. Additional file 5: Figure S2. Confirmation of tissue fractionation. Additional file 6: Figure S3. Annotation for rodent proteins.
