# Supplementary figures and images for: Differential effects of dopamine signalling on long-term memory formation and consolidation in rodent brain
Source: Proteome Sci. 2015 Mar 18;13:13. doi: 10.1186/s12953-015-0069-2 (PMC4387680; doi:10.1186/s12953-015-0069-2)

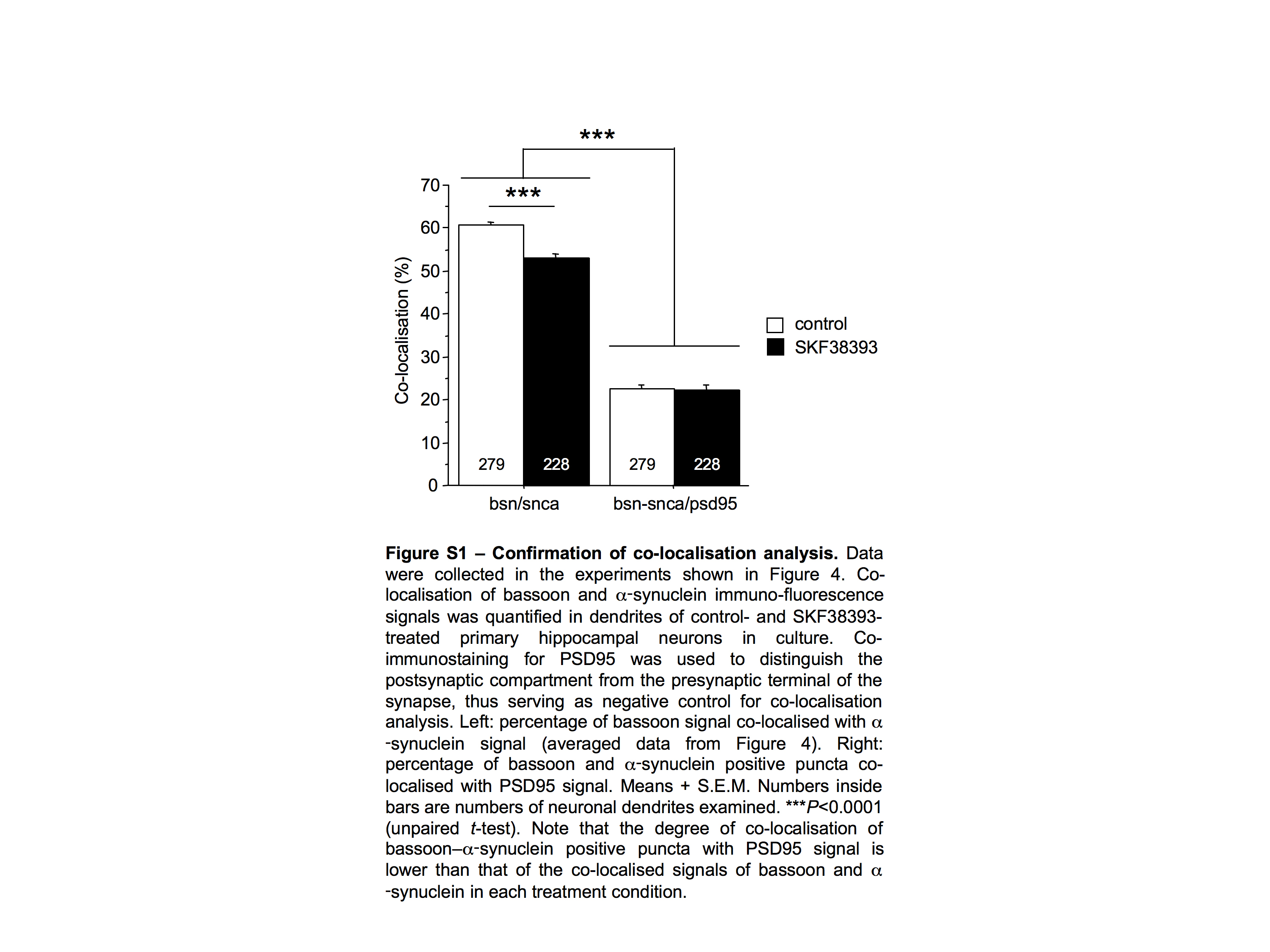

Supplement: Additional file 3: Figure S1. — Confirmation of co-localisation analysis. [file 12953_2015_69_MOESM3_ESM.jpeg]

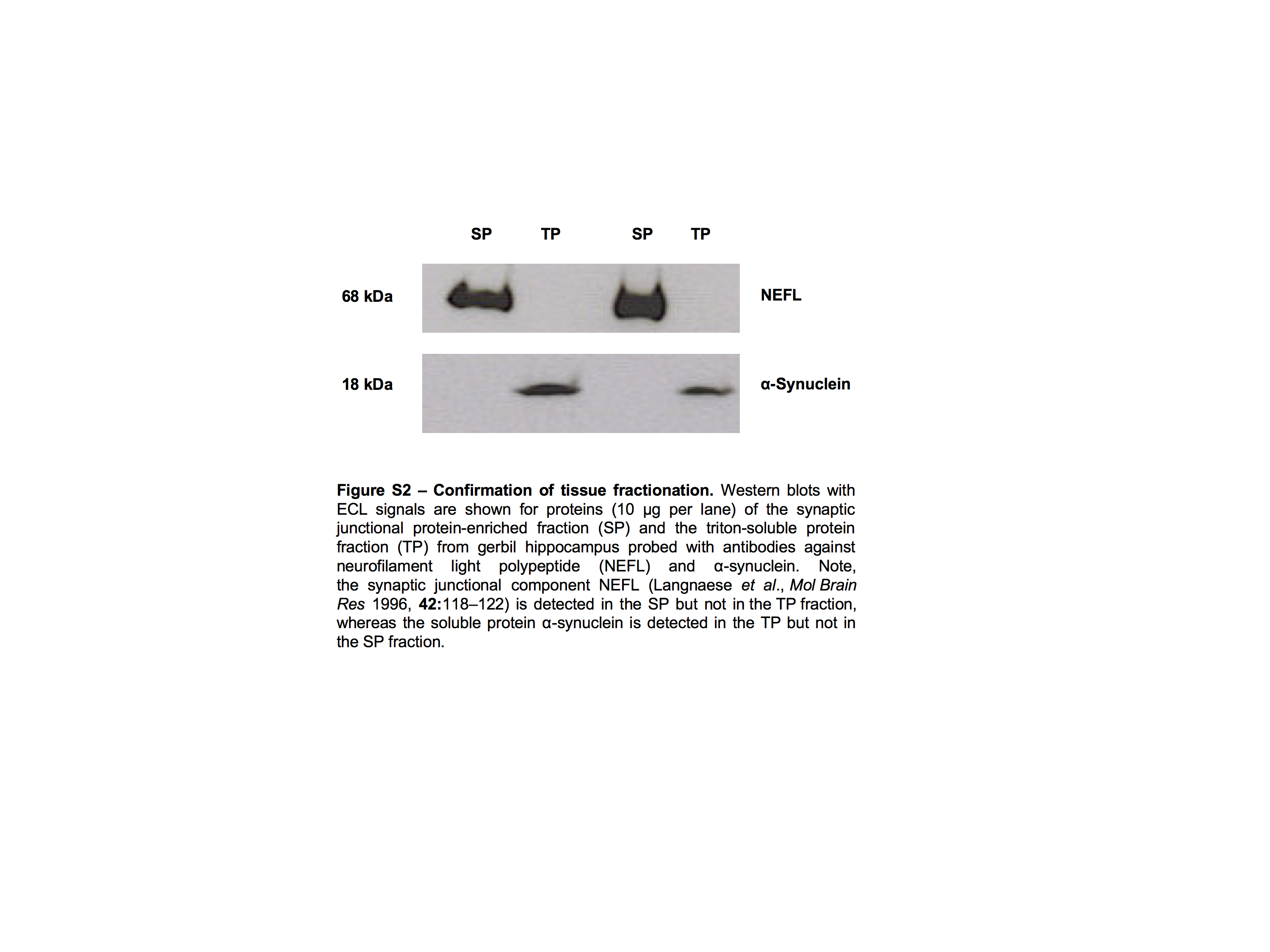

Supplement: Additional file 5: Figure S2. — Confirmation of tissue fractionation. [file 12953_2015_69_MOESM5_ESM.jpeg]

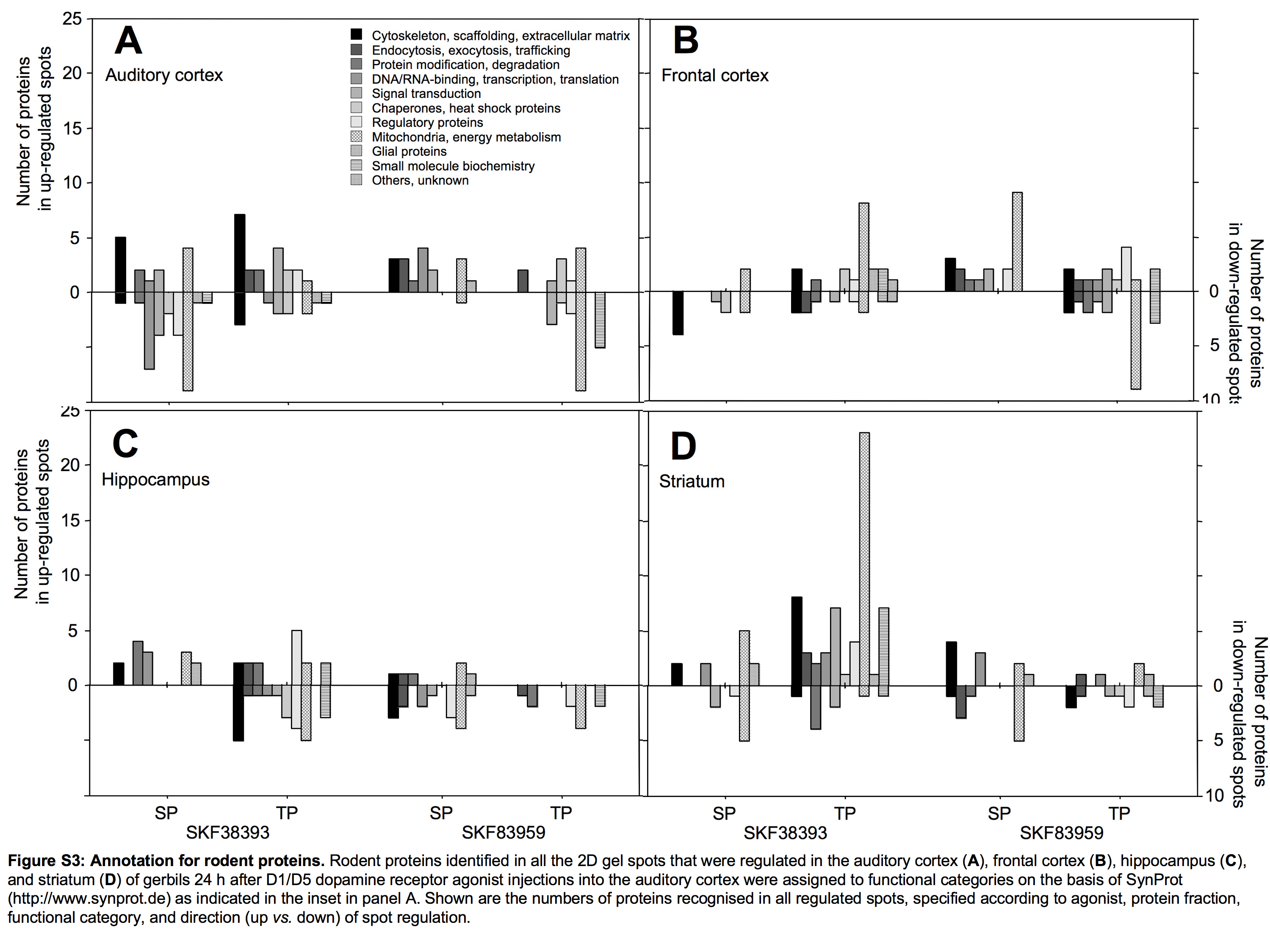

Supplement: Additional file 6: Figure S3. — Annotation for rodent proteins. [file 12953_2015_69_MOESM6_ESM.jpeg]
