# Supplementary material for: Differential effects of dopamine signalling on long-term memory formation and consolidation in rodent brain
Source: Proteome Sci. 2015 Mar 18;13:13. doi: 10.1186/s12953-015-0069-2 (PMC4387680; doi:10.1186/s12953-015-0069-2)
Supplement: Additional file 4: Tables S3-S10. — Behavioural data. [file 12953_2015_69_MOESM4_ESM.pdf]

Table S3. Values of repeated-measures ANOVA comparing behavioral measures over training sessions 1-16 across pharmacological treatment conditions and the indicated substrains.

	Main effect of treatment		Main effect of substrain		Main effect of session		Treatment x substrain		Treatment x session		Substrain x session		Treatment x substrain x session	
**Comparison across substrains C57BL/6JOlaHsd, C57BL/6JCrl, and C57BL/6JRccHsd**														
	$F_{5,84}$	P	$F_{2,84}$	P	$F_{15,1260}$	P	$F_{10,84}$	P	$F_{75,1260}$	P	$F_{30,1260}$	P	$F_{150,1260}$	P
CR^+	0.576	.7184	3.221	**.0449**	91.322	**<.0001**	1.463	.1680	1.332	**.0339**	5.909	**<.0001**	1.551	**<.0001**
CR^-	2.173	.0647	15.249	**<.0001**	37.366	**<.0001**	1.834	.0669	1.213	.1089	8.333	**<.0001**	1.313	**.0097**
D	0.565	.7262	0.455	.6362	188.616	**<.0001**	1.955	**.0488**	1.435	**.0103**	7.933	**<.0001**	1.378	**.0028**
**Comparison across substrains C57BL/6JOlaHsd and C57BL/6JCrl**														
	$F_{5,60}$	P	$F_{1,60}$	P	$F_{15,900}$	P	$F_{5,60}$	P	$F_{75,900}$	P	$F_{15,900}$	P	$F_{75,900}$	P
CR^+	1.074	.3840	3.557	.0642	70.583	**<.0001**	1.177	.3313	1.529	**.0035**	7.210	**<.0001**	1.689	**.0004**
CR^-	3.363	**.0096**	18.418	**<.0001**	18.161	**<.0001**	3.472	**.0080**	1.729	**.0002**	11.449	**<.0001**	0.972	.5464
D	1.675	.1545	0.199	.6570	135.040	**<.0001**	2.532	**.0381**	1.785	**<.0001**	15.314	**<.0001**	1.541	**.0030**
**Comparison across substrains C57BL/6JOlaHsd and C57BL/6JRccHsd**														
	$F_{5,58}$	P	$F_{1,58}$	P	$F_{15,870}$	P	$F_{5,58}$	P	$F_{75,870}$	P	$F_{15,870}$	P	$F_{75,870}$	P
CR^+	0.932	.4670	4.798	**.0325**	59.816	**<.0001**	2.078	.0811	1.485	**.0062**	9.311	**<.0001**	1.836	**<.0001**
CR^-	1.745	.1387	34.651	**<.0001**	23.312	**<.0001**	0.296	.9130	1.224	.1021	16.245	**<.0001**	1.725	**.0002**
D	0.984	.4353	0.819	.3692	103.681	**<.0001**	1.963	.0977	1.373	**.0230**	7.915	**<.0001**	1.778	**.0001**
**Comparison across substrains C57BL/6JCrl and C57BL/6JRccHsd**														
	$F_{5,50}$	P	$F_{1,50}$	P	$F_{15,750}$	P	$F_{5,50}$	P	$F_{75,750}$	P	$F_{15,750}$	P	$F_{75,750}$	P
CR^+	0.542	.7436	0.146	.7040	57.216	**<.0001**	1.024	.4138	1.195	.1331	1.675	.0510	1.153	.1859
CR^-	1.443	.2254	1.382	.2454	32.565	**<.0001**	1.725	.1460	0.941	.6204	1.485	.1042	1.260	.0751
D	0.528	.7540	0.265	.6092	139.437	**<.0001**	1.422	.2325	1.081	.3057	1.167	.2923	0.804	.8820

The relative frequencies of CR^+ and CR^- and the discrimination rate *D* were compared with 6 x 3 x 16 followed by 6 x 2 x 16 (pharmacological treatment x substrain x training session) repeated-measures ANOVA, with training session serving as the repeated measure. Significant values ($P<0.05$) in bold. Note that the two substrains of the 6J subpopulation, C57BL/6JCrl and C57BL/6JRccHsd, were indistinguishable from each other but differed significantly from C57BL/6JOlaHsd.

Table S4. Data recorded during FM discrimination training of vehicle-treated 6J (*n*=10) and 6JOla (*n*=7) mice.

Sub-population	Session	Relative frequency of CR+ [%]	Relative frequency of CR- [%]	Number of intertrial crossings
6J	1	31.67 ± 5.78	26.00 ± 5.11	3.30 ± 0.79
6J	2	40.00 ± 6.83	37.00 ± 7.59	2.10 ± 0.77
6J	3	37.33 ± 5.14	25.00 ± 6.35	2.00 ± 0.68
6J	4	34.00 ± 4.03	21.67 ± 6.07	1.60 ± 0.37
6J	5	38.33 ± 5.29	12.00 ± 4.19#	1.90 ± 0.80
6J	6	38.33 ± 8.02	8.67 ± 2.73#	2.10 ± 0.96
6J	7	44.33 ± 6.83	8.33 ± 3.42#	3.00 ± 1.63
6J	8	58.67 ± 4.82	5.33 ± 2.99#	2.20 ± 1.05
6J	9	69.67 ± 4.26	7.33 ± 3.21#	1.40 ± 0.50
6J	10	59.00 ± 6.52	9.67 ± 6.05#	2.30 ± 1.33
6J	11	68.00 ± 5.05	4.33 ± 2.81#	2.20 ± 1.36
6J	12	78.00 ± 4.45	9.00 ± 3.34#	2.70 ± 0.56
6J	13	77.67 ± 2.77	3.67 ± 1.95#	2.70 ± 0.98
6J	14	71.67 ± 3.45	9.33 ± 4.18#	3.20 ± 1.01
6J	15	69.67 ± 5.63	5.00 ± 2.00#	4.30 ± 2.66
6J	16	77.67 ± 3.07	4.67 ± 1.59#	2.60 ± 0.62
6JOla	1	9.52 ± 2.56*	8.57 ± 3.16*	1.57 ± 0.30
6JOla	2	29.52 ± 6.68	10.48 ± 3.21#*	2.00 ± 0.53
6JOla	3	40.95 ± 4.70	15.71 ± 4.16#	1.71 ± 0.61
6JOla	4	39.05 ± 6.81	13.81 ± 3.21#	1.29 ± 0.68
6JOla	5	46.19 ± 6.84	11.90 ± 6.50#	1.00 ± 0.31
6JOla	6	39.52 ± 4.57	8.10 ± 2.16#	0.86 ± 0.40
6JOla	7	62.86 ± 5.61	10.95 ± 4.35#	2.29 ± 0.78
6JOla	8	57.14 ± 6.60	2.38 ± 1.40#	1.71 ± 0.92
6JOla	9	69.52 ± 6.44	6.19 ± 1.53#	6.00 ± 3.08
6JOla	10	62.38 ± 3.54	8.57 ± 1.90#	3.00 ± 0.87
6JOla	11	60.95 ± 7.69	3.81 ± 1.53#	4.29 ± 2.20
6JOla	12	61.90 ± 5.29*	4.76 ± 2.71#	4.86 ± 1.37
6JOla	13	66.19 ± 6.23	7.62 ± 3.39#	5.14 ± 1.45
6JOla	14	69.05 ± 3.76	4.76 ± 1.23#	4.29 ± 0.92
6JOla	15	62.38 ± 5.43	8.10 ± 1.60#	5.29 ± 1.15
6JOla	16	64.76 ± 6.62	7.62 ± 3.62#	5.71 ± 1.97
Main effect of subpopulation		$F_{1,15}$=0.554 P=0.4682	$F_{1,15}$=1.250 P=0.2812	$F_{1,15}$=0.553 P=0.4685
Main effect of session		$F_{15,225}$=22.889 **P<0.0001**	$F_{15,225}$=6.541 **P<0.0001**	$F_{15,225}$=2.043 **P=0.0137**
Subpopulation x session		$F_{15,225}$=2.186 **P=0.0076**	$F_{15,225}$=2.889 **P=0.0003**	$F_{15,225}$=1.348 P=0.1751

Upper part: experimental data expressed as means ± SEM. Lower part: values of repeated-measures ANOVA over sessions across subpopulations. (*) P<0.05, significantly different from the corresponding value of 6J mice (two-sided unpaired *t*-test). (#) P<0.05, significantly different from the corresponding value of CR+ (two-sided paired *t*-test).

Table S5. Data recorded during FM discrimination training of SCH23390-treated 6J (*n*=12) and 6JOla (*n*=11) mice.

Sub-population	Session	Relative frequency of CR+ [%]	Relative frequency of CR- [%]	Number of intertrial crossings
6J	1	28.89 ± 3.29	28.33 ± 4.39	4.67 ± 0.78
6J	2	37.50 ± 5.58	30.00 ± 6.37	3.42 ± 1.03
6J	3	46.67 ± 7.86	31.39 ± 7.51	7.17 ± 3.07
6J	4	45.28 ± 7.63	23.33 ± 7.88#	3.42 ± 0.93
6J	5	48.33 ± 4.98	23.06 ± 6.69#	4.92 ± 1.64
6J	6	50.56 ± 6.18	15.00 ± 2.83#	7.50 ± 2.92
6J	7	63.06 ± 5.48	16.11 ± 3.67#	10.33 ± 3.46
6J	8	65.56 ± 5.95	17.50 ± 5.63#	9.75 ± 2.84
6J	9	66.11 ± 3.78	15.00 ± 3.16#	15.83 ± 7.43
6J	10	70.00 ± 6.33	13.33 ± 3.58#	19.83 ± 6.40
6J	11	66.94 ± 5.90	8.61 ± 2.83#	13.17 ± 3.63
6J	12	79.72 ± 3.47	8.06 ± 2.58#	19.83 ± 7.94
6J	13	71.94 ± 4.41	10.56 ± 3.50#	16.17 ± 5.45
6J	14	79.17 ± 3.90	6.94 ± 3.03#	22.17 ± 7.90
6J	15	80.00 ± 2.93	12.50 ± 4.55#	19.75 ± 7.36
6J	16	75.28 ± 4.11	10.00 ± 3.46#	20.75 ± 7.10
6JOla	1	9.70 ± 2.86*	12.12 ± 2.96*	2.91 ± 0.64
6JOla	2	23.94 ± 5.24	10.61 ± 4.04#*	0.73 ± 0.33
6JOla	3	27.88 ± 4.43	12.12 ± 2.51#*	2.55 ± 0.73
6JOla	4	39.39 ± 6.29	14.85 ± 5.46#	3.18 ± 1.88
6JOla	5	38.18 ± 6.20	10.61 ± 4.31#	5.82 ± 3.53
6JOla	6	37.88 ± 5.71	6.97 ± 2.36#*	3.91 ± 2.63
6JOla	7	40.30 ± 6.50*	9.39 ± 4.62#	3.91 ± 1.85
6JOla	8	44.55 ± 7.05*	3.64 ± 1.14#*	5.91 ± 2.81
6JOla	9	49.09 ± 5.52*	3.64 ± 1.38#*	4.64 ± 1.38
6JOla	10	63.33 ± 4.84	5.15 ± 1.58#	15.09 ± 7.22
6JOla	11	53.03 ± 4.89	6.06 ± 2.05#	7.45 ± 2.87
6JOla	12	62.12 ± 5.42*	9.39 ± 3.86#	10.00 ± 3.24
6JOla	13	68.18 ± 4.91	3.33 ± 1.85#	4.27 ± 1.18
6JOla	14	70.00 ± 4.14	4.85 ± 2.22#	6.18 ± 2.37
6JOla	15	65.15 ± 5.58*	6.36 ± 1.93#	6.45 ± 1.89
6JOla	16	64.55 ± 4.66	2.73 ± 0.99#	3.45 ± 0.99
Main effect of subpopulation		$F_{1,21}$=7.550 **P=0.0121**	$F_{1,21}$=8.439 **P=0.0085**	$F_{1,21}$=4.010 P=0.0583
Main effect of session		$F_{15,315}$=31.874 **P<0.0001**	$F_{15,315}$=4.901 **P<0.0001**	$F_{15,315}$=3.492 **P<0.0001**
Subpopulation x session		$F_{15,315}$=0.904 P=0.5604	$F_{15,315}$=1.404 P=0.1433	$F_{15,315}$=1.274 P=0.2165

Upper part: experimental data expressed as means ± SEM. Lower part: values of repeated-measures ANOVA over sessions across subpopulations. (*) P<0.05, significantly different from the corresponding value of 6J mice (two-sided unpaired *t*-test). (#) P<0.05, significantly different from the corresponding value of CR+ (two-sided paired *t*-test).

Table S6. Data recorded during FM discrimination training of SKF38393-treated 6J (*n*=15) and 6JOla (*n*=8) mice.

Sub-population	Session	Relative frequency of CR+ [%]	Relative frequency of CR- [%]	Number of intertrial crossings
6J	1	32.44 ± 3.50	22.67 ± 3.64#	8.73 ± 2.70
6J	2	45.33 ± 5.05	32.44 ± 5.48#	2.87 ± 0.49
6J	3	44.89 ± 5.39	29.33 ± 5.78#	2.07 ± 0.41
6J	4	29.78 ± 4.78	18.89 ± 5.27#	2.33 ± 0.53
6J	5	31.11 ± 6.42	9.33 ± 4.31#	2.33 ± 0.75
6J	6	34.67 ± 5.59	9.33 ± 5.91#	1.87 ± 0.67
6J	7	50.44 ± 5.64	8.00 ± 3.44#	3.00 ± 0.72
6J	8	54.44 ± 4.89	7.11 ± 3.25#	2.67 ± 0.85
6J	9	62.89 ± 5.85	7.11 ± 2.15#	2.87 ± 0.92
6J	10	65.33 ± 5.03	6.44 ± 2.26#	2.87 ± 0.68
6J	11	64.89 ± 5.55	4.00 ± 1.09#	4.60 ± 2.95
6J	12	70.67 ± 3.97	3.56 ± 1.40#	4.60 ± 1.71
6J	13	80.22 ± 3.18	5.11 ± 2.00#	5.60 ± 2.78
6J	14	73.11 ± 3.18	4.89 ± 1.78#	3.93 ± 0.85
6J	15	80.22 ± 3.21	13.11 ± 7.04#	3.20 ± 0.89
6J	16	73.78 ± 3.10	5.56 ± 2.38#	2.20 ± 0.85
6JOla	1	8.75 ± 2.27*	5.42 ± 2.88*	0.50 ± 0.38
6JOla	2	20.83 ± 5.34*	0.83 ± 0.55#*	0.12 ± 0.12
6JOla	3	44.58 ± 8.52	3.75 ± 1.33#*	1.25 ± 0.62
6JOla	4	50.42 ± 9.33*	5.42 ± 2.59#	1.00 ± 0.38
6JOla	5	48.75 ± 9.41	3.33 ± 1.67#	0.75 ± 0.49
6JOla	6	49.58 ± 9.22	2.92 ± 0.98#	1.38 ± 0.56
6JOla	7	60.83 ± 6.51	4.58 ± 2.81#	2.38 ± 1.13
6JOla	8	53.33 ± 6.24	3.33 ± 1.41#	1.75 ± 0.70
6JOla	9	59.17 ± 6.51	1.25 ± 0.88#	0.75 ± 0.41
6JOla	10	49.17 ± 4.07*	2.50 ± 2.50#	3.25 ± 2.11
6JOla	11	49.58 ± 10.36	0.00 ± 0.00#*	0.50 ± 0.27
6JOla	12	44.58 ± 9.41*	2.50 ± 0.83#	2.88 ± 1.47
6JOla	13	40.42 ± 5.65*	5.42 ± 2.27#	4.12 ± 1.06
6JOla	14	42.92 ± 7.08*	1.67 ± 1.26#	3.62 ± 1.24
6JOla	15	50.83 ± 5.93*	1.67 ± 1.09#	1.50 ± 0.46
6JOla	16	62.08 ± 6.01	2.50 ± 1.37#	3.88 ± 2.18
Main effect of subpopulation		$F_{1,21}$=3.513 P=0.0749	$F_{1,21}$=7.145 P=0.0142	$F_{1,21}$=2.126 P=0.1596
Main effect of session		$F_{15,315}$=14.458 **P<0.0001**	$F_{15,315}$=4.007 **P=<0.0001**	$F_{15,315}$=1.383 P=0.1538
Subpopulation x session		$F_{15,315}$=7,762 **P<0.0001**	$F_{15,315}$=3.584 **P<0.0001**	$F_{15,315}$=1.365 P=0.1629

Upper part: experimental data expressed as means ± SEM. Lower part: values of repeated-measures ANOVA over sessions across subpopulations. (*) P<0.05, significantly different from the corresponding value of 6J mice (two-sided unpaired *t*-test). (#) P<0.05, significantly different from the corresponding value of CR+ (two-sided paired *t*-test).

Table S7. Data recorded during FM discrimination training of SKF83822-treated 6J (n=13) and 6JOla (n=7) mice.

Sub-population	Session	Relative frequency of CR+ [%]	Relative frequency of CR- [%]	Number of intertrial crossings
6J	1	33.33 ± 6.00	29.23 ± 5.87	4.92 ± 1.51
6J	2	40.51 ± 5.25	41.54 ± 6.63	4.92 ± 1.91
6J	3	46.67 ± 6.53	40.00 ± 6.70	8.15 ± 3.94
6J	4	28.46 ± 5.29	27.95 ± 6.78	8.08 ± 4.73
6J	5	36.92 ± 5.45	25.90 ± 7.18	22.23 ± 16.54
6J	6	37.44 ± 8.63	13.08 ± 3.67#	13.15 ± 8.86
6J	7	39.49 ± 7.29	11.03 ± 2.49#	11.54 ± 4.51
6J	8	58.46 ± 6.84	13.08 ± 2.49#	14.62 ± 8.90
6J	9	63.33 ± 4.70	15.38 ± 3.63#	17.08 ± 11.12
6J	10	59.74 ± 6.61	10.00 ± 1.89#	10.62 ± 2.62
6J	11	69.49 ± 3.85	10.51 ± 2.77#	7.54 ± 3.05
6J	12	69.23 ± 7.37	11.28 ± 2.81#	11.08 ± 2.09
6J	13	73.08 ± 4.96	13.85 ± 4.60#	11.54 ± 4.13
6J	14	78.21 ± 2.99	8.97 ± 2.73#	21.46 ± 10.33
6J	15	78.72 ± 3.63	14.36 ± 4.76#	12.54 ± 7.03
6J	16	82.31 ± 2.83	11.54 ± 3.22#	9.38 ± 2.40
6JOla	1	17.14 ± 5.46	1.90 ± 0.99#*	1.00 ± 0.38
6JOla	2	28.57 ± 9.28	4.76 ± 2.28#*	2.29 ± 1.67
6JOla	3	42.38 ± 12.06	3.33 ± 1.63#*	1.43 ± 0.81
6JOla	4	46.67 ± 11.84	0.95 ± 0.61#*	1.43 ± 1.27
6JOla	5	61.43 ± 11.12*	1.43 ± 0.99#*	1.71 ± 0.81
6JOla	6	63.33 ± 4.48	3.81 ± 1.35#	2.71 ± 1.25
6JOla	7	61.90 ± 9.73	2.86 ± 1.35#*	3.00 ± 1.54
6JOla	8	67.62 ± 8.25	5.71 ± 2.02#	5.29 ± 2.36
6JOla	9	75.24 ± 7.20	5.24 ± 3.40#	2.86 ± 0.86
6JOla	10	70.00 ± 6.98	4.29 ± 2.27#	3.00 ± 0.87
6JOla	11	73.33 ± 6.78	3.33 ± 1.26#	3.43 ± 1.46
6JOla	12	72.86 ± 5.11	5.24 ± 2.61#	7.14 ± 3.84
6JOla	13	71.43 ± 8.00	0.95 ± 0.61#	4.57 ± 1.72
6JOla	14	69.52 ± 9.69	3.33 ± 1.78#	4.43 ± 2.27
6JOla	15	80.95 ± 4.81	1.43 ± 0.67#	5.43 ± 2.64
6JOla	16	84.76 ± 2.50	4.29 ± 2.69#	3.14 ± 0.96
Main effect of subpopulation		$F_{1,18}$=0.897 P=0.3562	$F_{1,18}$=22.528 **P=0.0002**	$F_{1,18}$=1.704 P=0.2082
Main effect of session		$F_{15,270}$=18.767 **P<0.0001**	$F_{15,270}$=3.306 **P<0.0001**	$F_{15,270}$=0.546 P=0.9127
Subpopulation x session		$F_{15,270}$=2.535 **P=0.0015**	$F_{15,270}$=3.669 **P<0.0001**	$F_{15,270}$=0.405 P=0.9772

Upper part: experimental data expressed as means ± SEM. Lower part: values of repeated-measures ANOVA over sessions across subpopulations. (*) P<0.05, significantly different from the corresponding value of 6J mice (two-sided unpaired t-test). (#) P<0.05, significantly different from the corresponding value of CR+ (two-sided paired t-test).

Table S8. Data recorded during FM discrimination training of SKF83959-treated 6J (*n*=6) and 6JOla (*n*=3) mice.

Sub-population	Session	Relative frequency of CR+ [%]	Relative frequency of CR- [%]	Number of intertrial crossings
6J	1	37.78 ± 4.27	37.22 ± 4.98	8.17 ± 1.92
6J	2	64.44 ± 7.78	59.44 ± 11.59	4.50 ± 1.18
6J	3	56.11 ± 11.33	51.67 ± 15.46	4.50 ± 1.84
6J	4	51.11 ± 12.01	43.89 ± 19.12	10.67 ± 5.94
6J	5	38.33 ± 5.95	25.00 ± 13.71	29.83 ± 19.73
6J	6	30.00 ± 7.70	14.44 ± 9.38	20.17 ± 13.14
6J	7	50.00 ± 7.79	15.00 ± 7.24#	18.67 ± 7.88
6J	8	53.89 ± 10.31	12.78 ± 4.82#	33.50 ± 18.19
6J	9	57.78 ± 8.97	9.44 ± 3.15#	11.33 ± 4.97
6J	10	65.56 ± 4.44	9.44 ± 2.64#	19.50 ± 10.08
6J	11	67.78 ± 6.70	3.89 ± 1.02#	11.17 ± 4.96
6J	12	74.44 ± 3.18	4.44 ± 2.68#	24.00 ± 10.19
6J	13	69.44 ± 3.49	7.22 ± 2.00#	17.67 ± 7.15
6J	14	74.44 ± 5.07	6.67 ± 1.49#	17.00 ± 5.30
6J	15	85.56 ± 3.30	10.56 ± 3.69#	15.83 ± 5.72
6J	16	75.56 ± 5.49	3.89 ± 1.59#	6.17 ± 1.58
6JOla	1	7.78 ± 4.01*	4.44 ± 2.94*	1.67 ± 1.67
6JOla	2	15.56 ± 4.44*	24.44 ± 2.22	1.33 ± 0.88
6JOla	3	30.00 ± 1.92	22.22 ± 5.56	1.33 ± 0.33
6JOla	4	27.78 ± 2.22	16.67 ± 9.62	2.00 ± 1.15
6JOla	5	24.44 ± 6.19	17.78 ± 7.29	2.00 ± 0.58
6JOla	6	32.22 ± 9.49	10.00 ± 3.85	2.00 ± 1.00
6JOla	7	42.22 ± 2.22	11.11 ± 1.11#	3.33 ± 1.86
6JOla	8	67.78 ± 10.60	6.67 ± 0.00#	11.67 ± 6.06
6JOla	9	62.22 ± 11.76	8.89 ± 2.94#	3.00 ± 0.58
6JOla	10	64.44 ± 5.88	10.00 ± 1.92#	9.00 ± 5.86
6JOla	11	45.56 ± 11.28	17.78 ± 6.19*	2.00 ± 0.58
6JOla	12	47.78 ± 17.46	0.00 ± 0.00	1.67 ± 1.67
6JOla	13	54.44 ± 11.28	13.33 ± 13.33#	2.67 ± 0.33
6JOla	14	55.56 ± 7.78	16.67 ± 3.85*	4.67 ± 1.45
6JOla	15	55.56 ± 7.78*	2.22 ± 1.11#	4.33 ± 1.45
6JOla	16	48.89 ± 5.88*	1.11 ± 1.11#	6.33 ± 4.84
Main effect of subpopulation		$F_{1,7}$=7.390 **P=0.0298**	$F_{1,7}$=1.329 P=0.2868	$F_{1,7}$=3.294 P=0.1124
Main effect of session		$F_{15,105}$=7.632 **P<0.0001**	$F_{15,105}$=4.398 **P<0.0001**	$F_{15,105}$=0.696 P=0.7832
Subpopulation x session		$F_{15,105}$=2.248 **P=0.0088**	$F_{15,105}$=1.805 **P=0.0432**	$F_{15,105}$=0.389 P=0.9794

Upper part: experimental data expressed as means ± SEM. Lower part: values of repeated-measures ANOVA over sessions across subpopulations. (*) P<0.05, significantly different from the corresponding value of 6J mice (two-sided unpaired *t*-test). (#) P<0.05, significantly different from the corresponding value of CR+ (two-sided paired *t*-test).

Table S9. Data recorded during FM discrimination training of 6J (*n*=6) and 6JOla (*n*=4) mice treated with a combination of SKF83822 and SKF83959.

Sub-population	Session	Relative frequency of		Number of intertrial crossings
		CR+ [%]	CR- [%]	
6J	1	26.67 ± 10.51	26.67 ± 12.02	4.67 ± 1.43
6J	2	40.56 ± 13.06	38.89 ± 14.37	3.33 ± 1.15
6J	3	40.00 ± 12.62	32.78 ± 17.14	1.00 ± 0.52
6J	4	25.56 ± 7.63	20.56 ± 9.79	3.00 ± 1.37
6J	5	30.00 ± 8.07	12.22 ± 7.78	1.33 ± 0.56
6J	6	38.33 ± 9.02	5.00 ± 2.24#	6.33 ± 4.62
6J	7	56.67 ± 5.51	4.44 ± 1.65#	2.33 ± 1.02
6J	8	68.33 ± 5.82	14.44 ± 5.28#	3.50 ± 1.69
6J	9	60.00 ± 3.55	6.11 ± 4.25#	2.50 ± 1.45
6J	10	78.89 ± 4.10	7.22 ± 5.33#	2.67 ± 1.50
6J	11	73.89 ± 6.23	2.78 ± 1.34#	2.83 ± 1.56
6J	12	75.56 ± 2.68	3.33 ± 1.22#	2.67 ± 1.48
6J	13	78.89 ± 6.13	5.56 ± 2.22#	2.83 ± 2.07
6J	14	68.89 ± 6.36	2.78 ± 1.02#	2.17 ± 1.08
6J	15	78.33 ± 4.93	6.11 ± 2.18#	8.67 ± 5.28
6J	16	71.67 ± 8.33	20.00 ± 5.96#	4.83 ± 1.49
6JOla	1	6.67 ± 3.33	2.50 ± 0.83	1.25 ± 0.75
6JOla	2	36.67 ± 13.81	2.50 ± 1.60	0.75 ± 0.48
6JOla	3	32.50 ± 13.77	1.67 ± 0.96	0.00 ± 0.00
6JOla	4	41.67 ± 12.21	1.67 ± 1.67#	0.25 ± 0.25
6JOla	5	65.83 ± 7.25*	7.50 ± 3.15#	2.50 ± 0.87
6JOla	6	77.50 ± 7.12*	8.33 ± 0.96#	2.00 ± 1.35
6JOla	7	66.67 ± 4.91	4.17 ± 2.50#	2.75 ± 0.95
6JOla	8	65.00 ± 17.56	5.83 ± 3.70#	2.75 ± 0.75
6JOla	9	61.67 ± 6.45	4.17 ± 2.50#	3.50 ± 1.55
6JOla	10	78.33 ± 4.81	15.83 ± 5.99#	4.25 ± 1.11
6JOla	11	63.33 ± 3.60	6.67 ± 3.04#	2.00 ± 0.91
6JOla	12	65.83 ± 6.29	9.17 ± 2.50#*	4.00 ± 0.71
6JOla	13	67.50 ± 8.75	10.00 ± 1.92#	6.00 ± 3.11
6JOla	14	59.17 ± 10.22	4.17 ± 3.15#	1.50 ± 0.96
6JOla	15	60.00 ± 8.28	10.00 ± 4.30#	4.00 ± 1.35
6JOla	16	66.67 ± 6.80	8.33 ± 2.15#	5.00 ± 1.08
Main effect of subpopulation		$F_{1,8}$=0.001<br>*P*=0.9761	$F_{1,8}$=1.514<br>*P*=0.2535	$F_{1,8}$=0.238<br>*P*=0.6385
Main effect of session		$F_{15,120}$=9.794<br>***P*<0.0001**	$F_{15,120}$=1.372<br>*P*=0.1722	$F_{15,120}$=1.304<br>*P*=0.2102
Subpopulation x session		$F_{15,120}$=2.395<br>***P*=0.0046**	$F_{15,120}$=2.735<br>***P*=0.0012**	$F_{15,120}$=0.818<br>*P*=0.6563

Upper part: experimental data expressed as means ± SEM. Lower part: values of repeated-measures ANOVA over sessions across subpopulations. (*) $P<0.05$, significantly different from the corresponding value of 6J mice (two-sided unpaired *t*-test). (#) $P<0.05$, significantly different from the corresponding value of CR+ (two-sided paired *t*-test).

Table S10. Values of within-session ANOVA comparing discrimination rates *D* across pharmacological treatment conditions and subpopulations 6JOla and 6J.

Session	Main effect of treatment		Main effect of subpopulation		Treatment x subpopulation	
	$F_{5,90}$	P	$F_{1,90}$	P	$F_{5,90}$	P
1	1.939	.0956	0.064	.8015	1.400	.2319
2	1.962	.0919	12.411	**.0007**	2.892	**.0181**
3	1.562	.1789	11.990	**.0008**	1.545	.1841
4	0.943	.4570	21.187	**<.0001**	2.799	**.0214**
5	1.866	.1081	14.761	**.0002**	3.296	**.0089**
6	2.212	.0599	9.972	**.0022**	2.326	**.0491**
7	1.489	.2012	2.842	.0953	2.460	**.0389**
8	0.587	.7095	1.540	.2178	0.707	.6197
9	1.258	.2880	1.137	.2892	1.039	.3999
10	0.932	.4644	0.002	.9667	1.147	.3416
11	1.467	.2085	6.297	**.0139**	1.605	.1669
12	0.549	.7389	9.539	**.0027**	1.644	.1564
13	1.544	.1841	11.017	**.0013**	5.213	**.0003**
14	2.497	**.0364**	12.053	**.0008**	2.263	.0548
15	1.391	.2352	7.839	**.0063**	2.419	**.0418**
16	2.999	**.0150**	3.266	.0741	2.349	**.0472**

Significant values (P<0.05) in bold.
